# Supplementary material for: A TOPBP1 allele causing male infertility uncouples XY silencing dynamics from sex body formation
Source: eLife. 2024 Feb 23;12:RP90887. doi: 10.7554/eLife.90887 (PMC10942628; doi:10.7554/eLife.90887)
Supplement: Figure 2—source data 1. [file elife-90887-fig2-data1.zip › Figure 2_source_data/Figure 2 Source Data File titles.docx]

Figure 2 Source Data File titles

Figure 2—source data 1. Original file for the western blot analysis in Figure 2E (anti-CHK1, anti-CHK2, and anti-GAPDH).

Figure 2—source data 2. PDF containing Figure 2E and original scans of the relevant western blot analysis (anti-CHK1, anti-CHK2, and anti-GAPDH) with highlighted bands.

Figure 2—source data 3.Original file for the western blot analysis in Figure 2E (anti-KAP1).

Figure 2—source data 4.PDF containing Figure 2E and original scans of the relevant western blot analysis (anti-KAP1) with highlighted bands.

Figure 2—source data 5.Original file for the western blot analysis in Figure 2E (anti-RPA).

Figure 2—source data 6.PDF containing Figure 2E and original scans of the relevant western blot analysis (anti-RPA) with highlighted bands.

Figure 2—source data 7.Original file for the western blot analysis in Figure 2E (anti-phospho KAP1-S824).

Figure 2—source data 8.PDF containing Figure 2E and original scans of the relevant western blot analysis (anti-phospho KAP1-S824) with highlighted bands.

Figure 2—source data 9.Original file for the western blot analysis in Figure 2E (anti-phospho CHK1-S345).

Figure 2—source data 10.PDF containing Figure 2E and original scans of the relevant western blot analysis (anti-phospho CHK1-S345) with highlighted bands.

Figure 2—source data 11.Original file for the western blot analysis in Figure 2E (anti-phospho CHK1-S317).

Figure 2—source data 12.PDF containing Figure 2E and original scans of the relevant western blot analysis (anti-phospho CHK1-S317) with highlighted bands.

Figure 2—source data 13.Original file for the western blot analysis in Figure 2E (anti-phospho RPA-S4/S8).

Figure 2—source data 14.PDF containing Figure 2E and original scans of the relevant western blot analysis (anti-phospho RPA-S4/S8) with highlighted bands.
