# Supplementary figures and images for: A TOPBP1 allele causing male infertility uncouples XY silencing dynamics from sex body formation
Source: eLife. 2024 Feb 23;12:RP90887. doi: 10.7554/eLife.90887 (PMC10942628; doi:10.7554/eLife.90887)

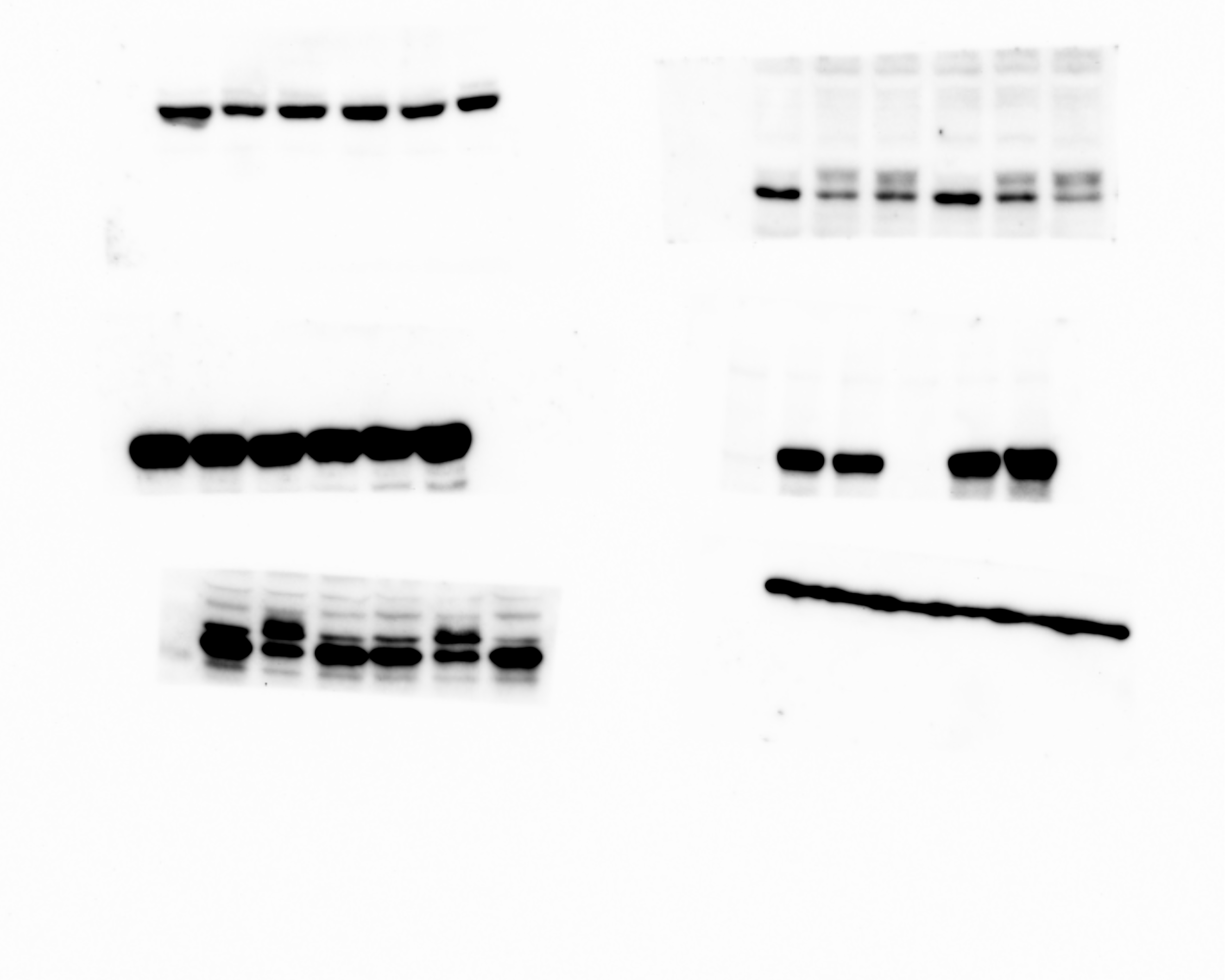

Supplement: Figure 2—source data 1. [file elife-90887-fig2-data1.zip › Figure 2_source_data/Figure_2_source_1.tif]

Figure 2 Source Data 10

E

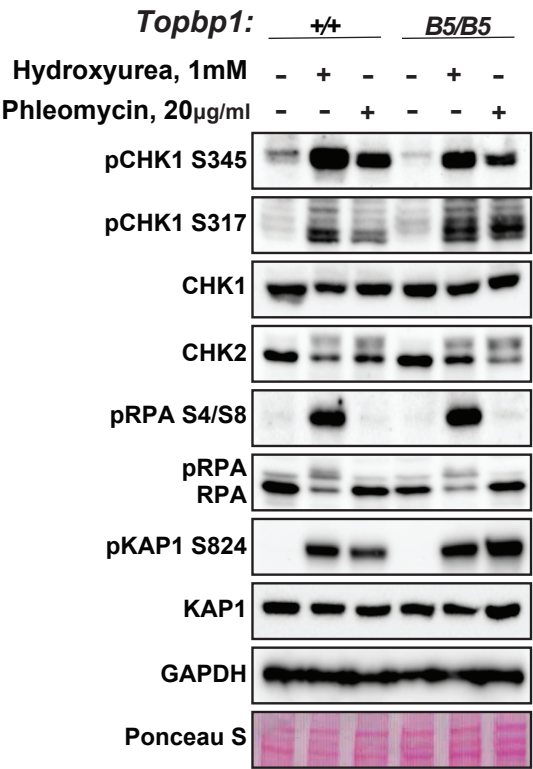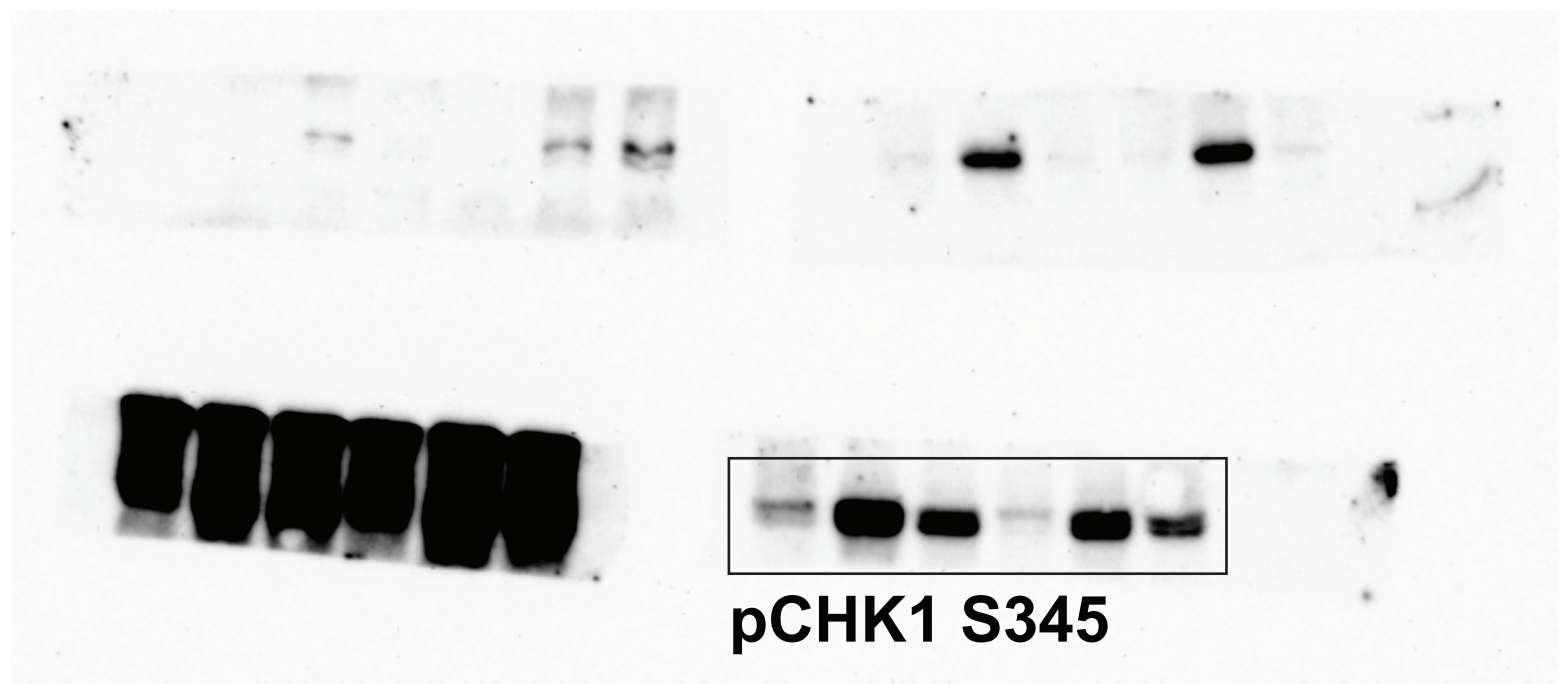

Supplement: Figure 2—source data 1. [file elife-90887-fig2-data1.zip › Figure 2_source_data/Figure_2_source_10.pdf]

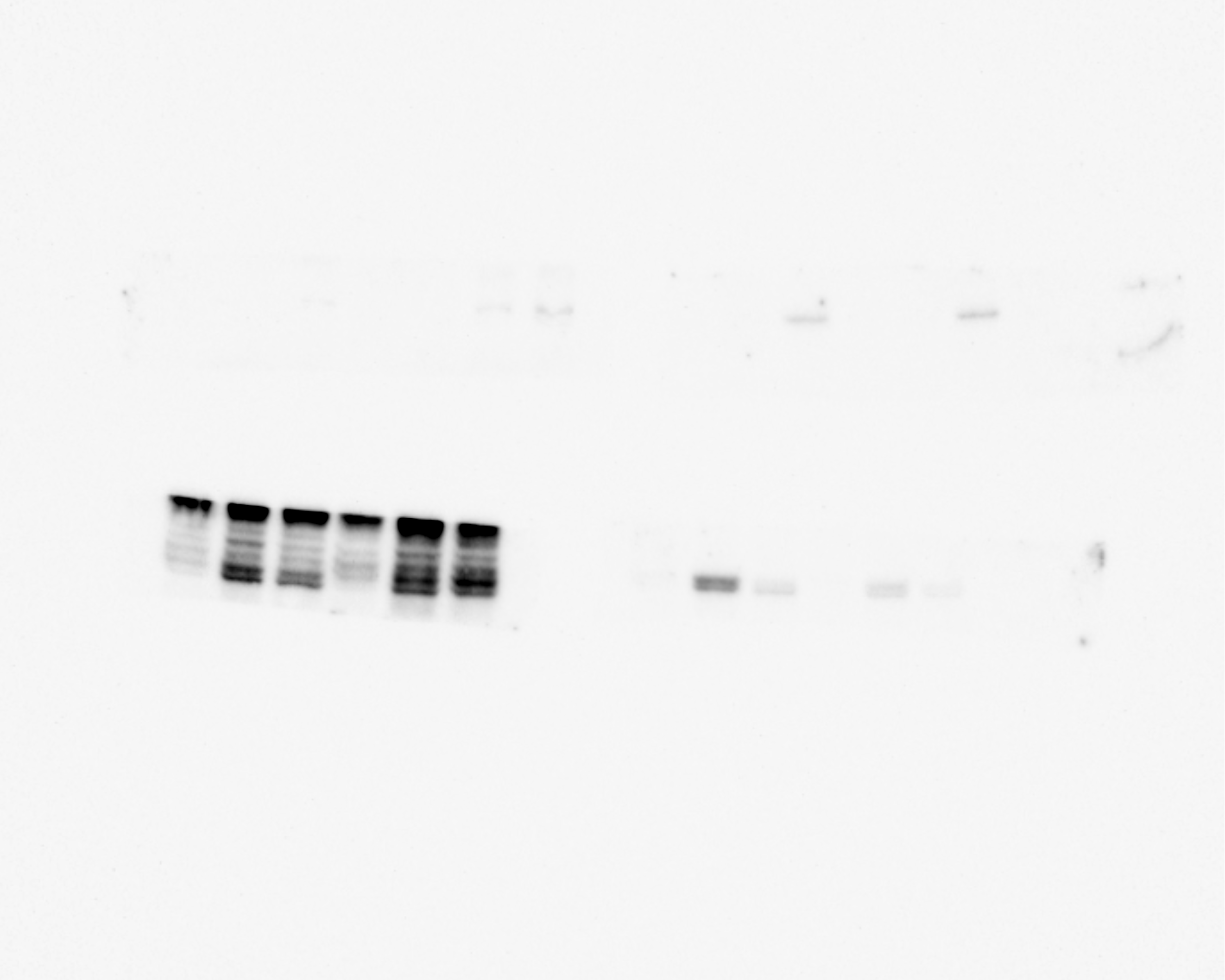

Supplement: Figure 2—source data 1. [file elife-90887-fig2-data1.zip › Figure 2_source_data/Figure_2_source_11.tif]

Figure 2 Source Data 12

E

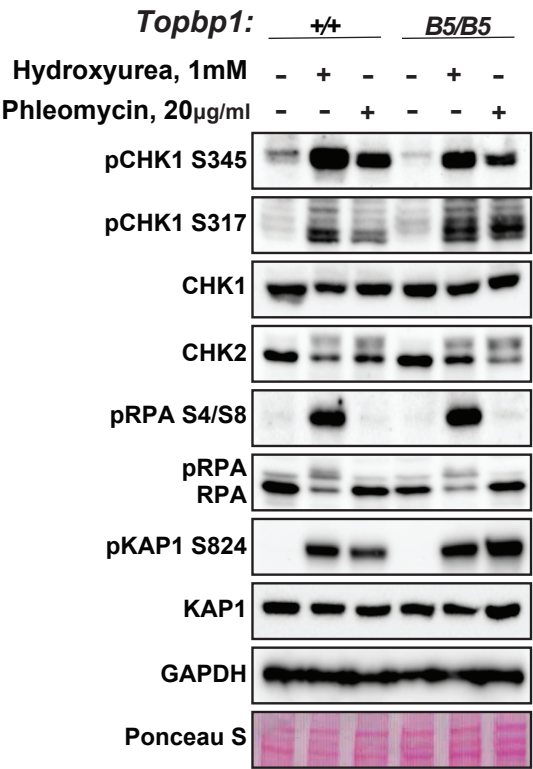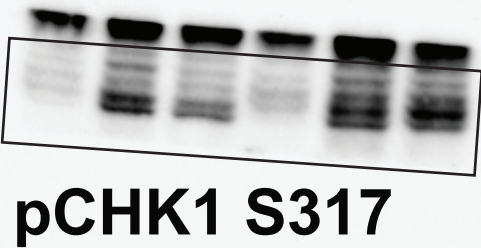

Supplement: Figure 2—source data 1. [file elife-90887-fig2-data1.zip › Figure 2_source_data/Figure_2_source_12.pdf]

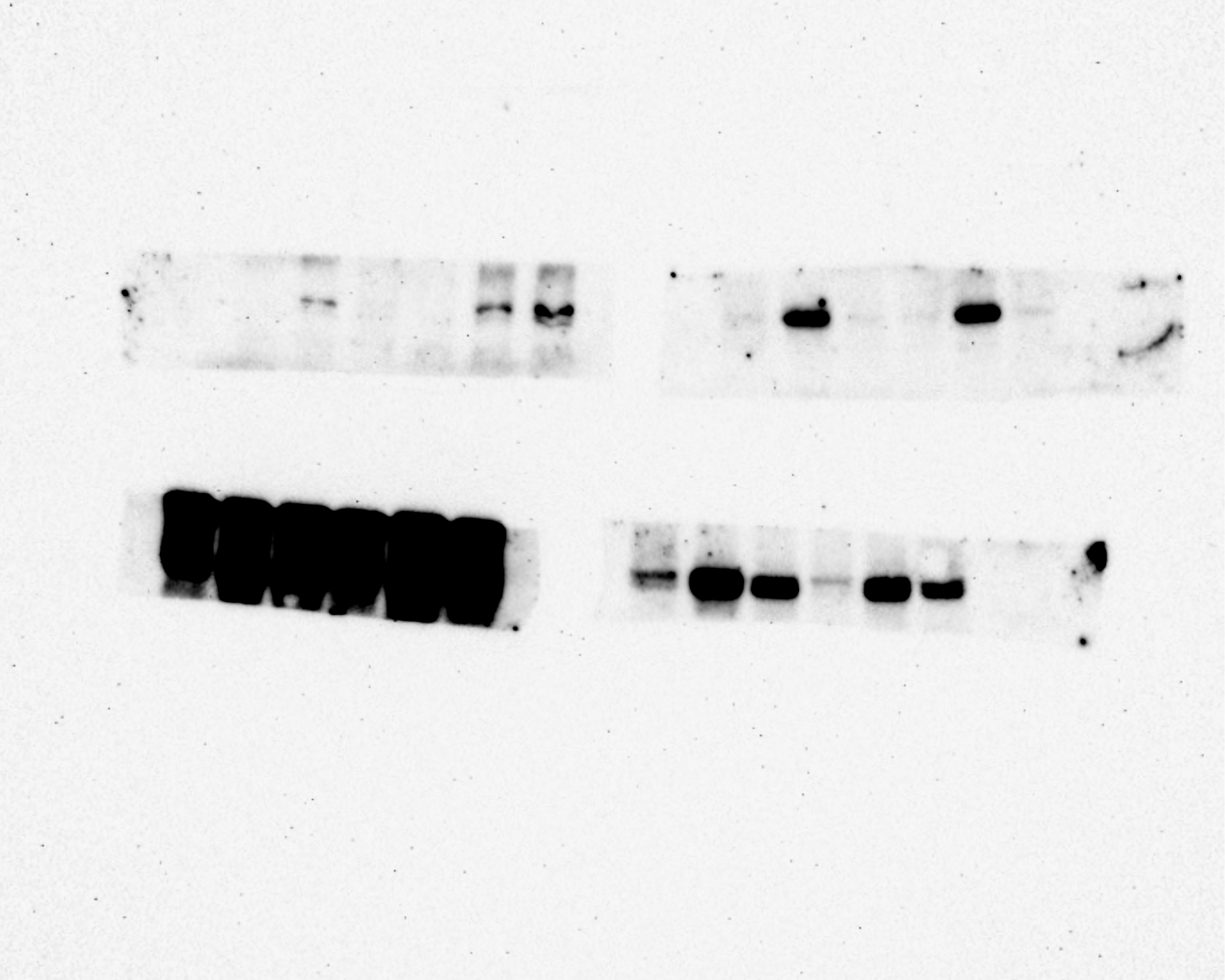

Supplement: Figure 2—source data 1. [file elife-90887-fig2-data1.zip › Figure 2_source_data/Figure_2_source_13.tif]

Figure 2 Source Data 14

E

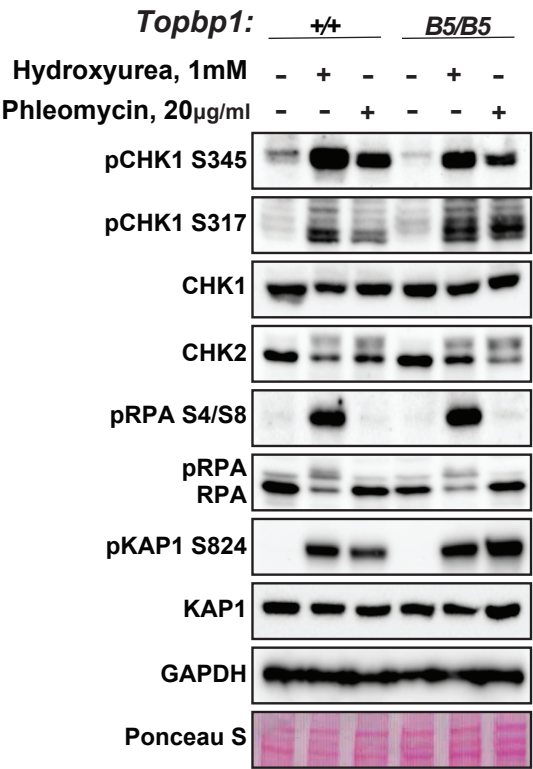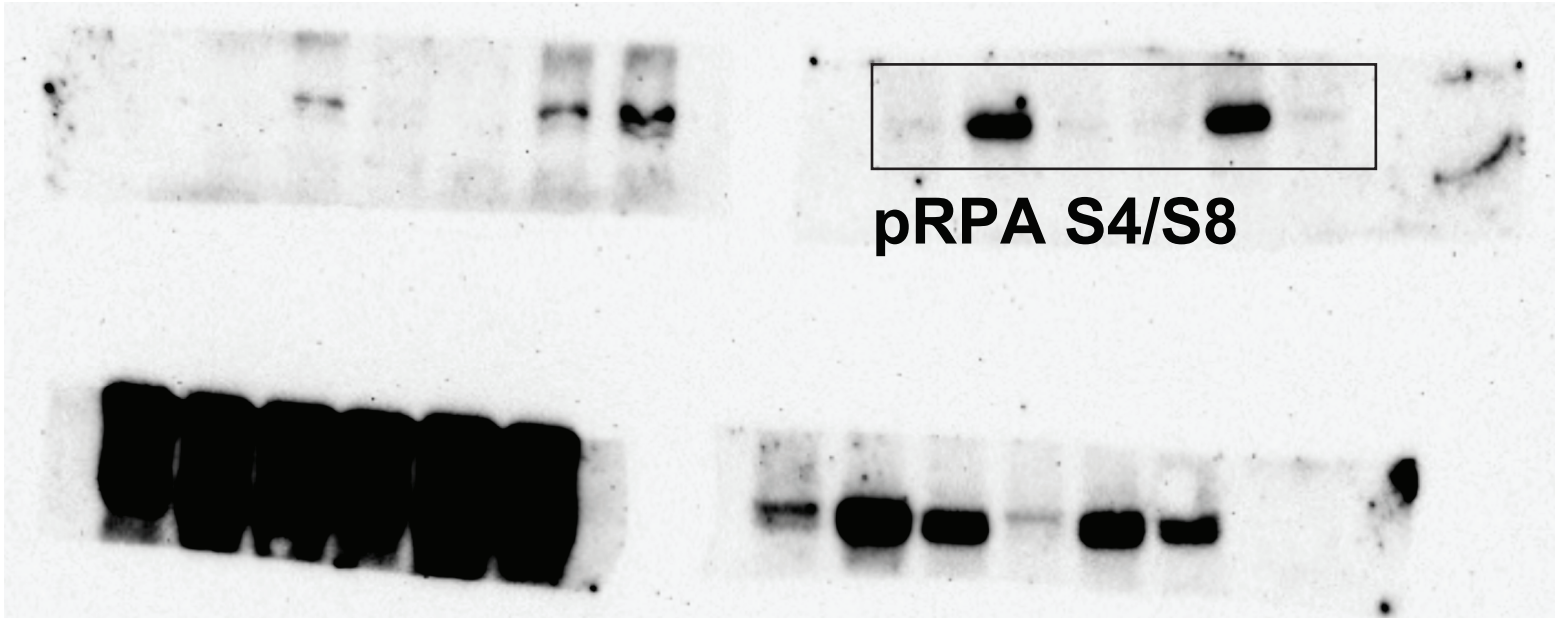

Supplement: Figure 2—source data 1. [file elife-90887-fig2-data1.zip › Figure 2_source_data/Figure_2_source_14.pdf]

Figure 2 Source Data 2

E

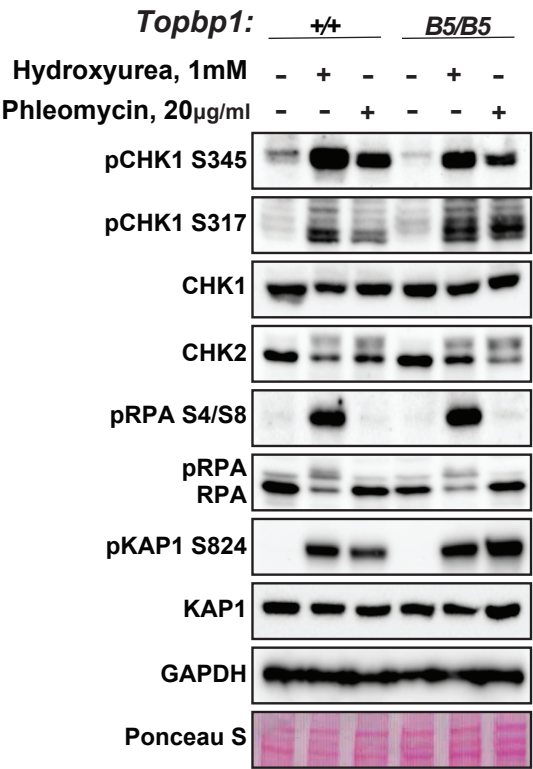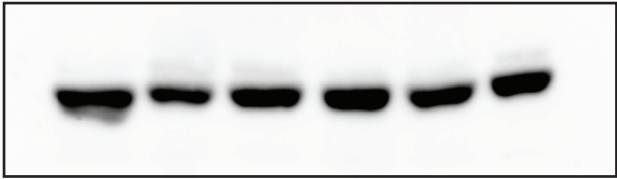

CHK1

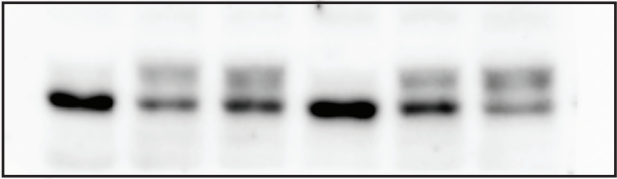

CHK2

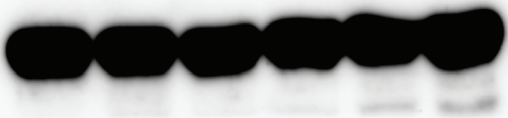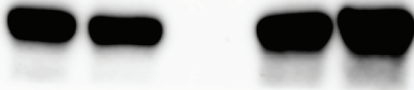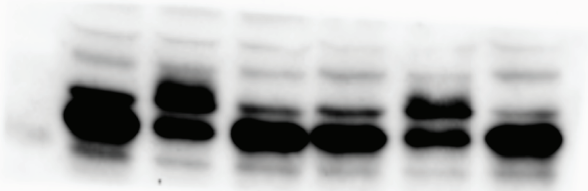

GAPDH

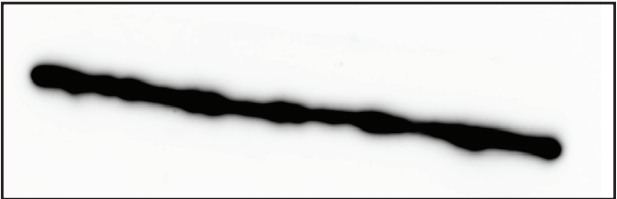

Supplement: Figure 2—source data 1. [file elife-90887-fig2-data1.zip › Figure 2_source_data/Figure_2_source_2.pdf]

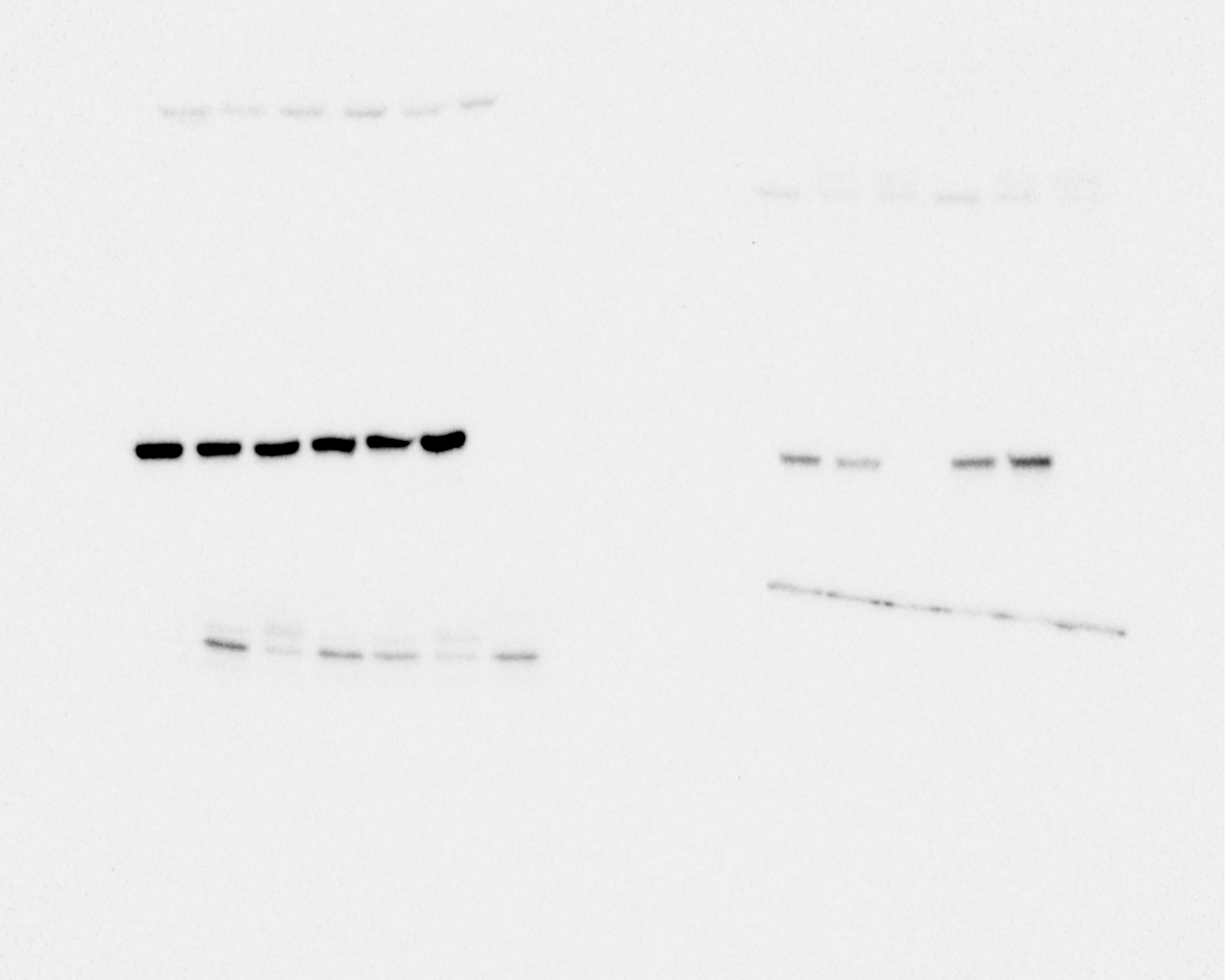

Supplement: Figure 2—source data 1. [file elife-90887-fig2-data1.zip › Figure 2_source_data/Figure_2_source_3.tif]

Figure 2 Source Data 4

E

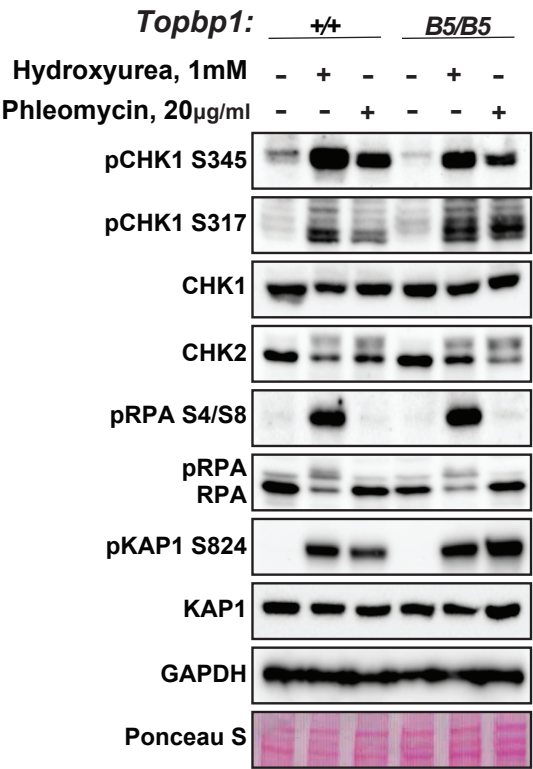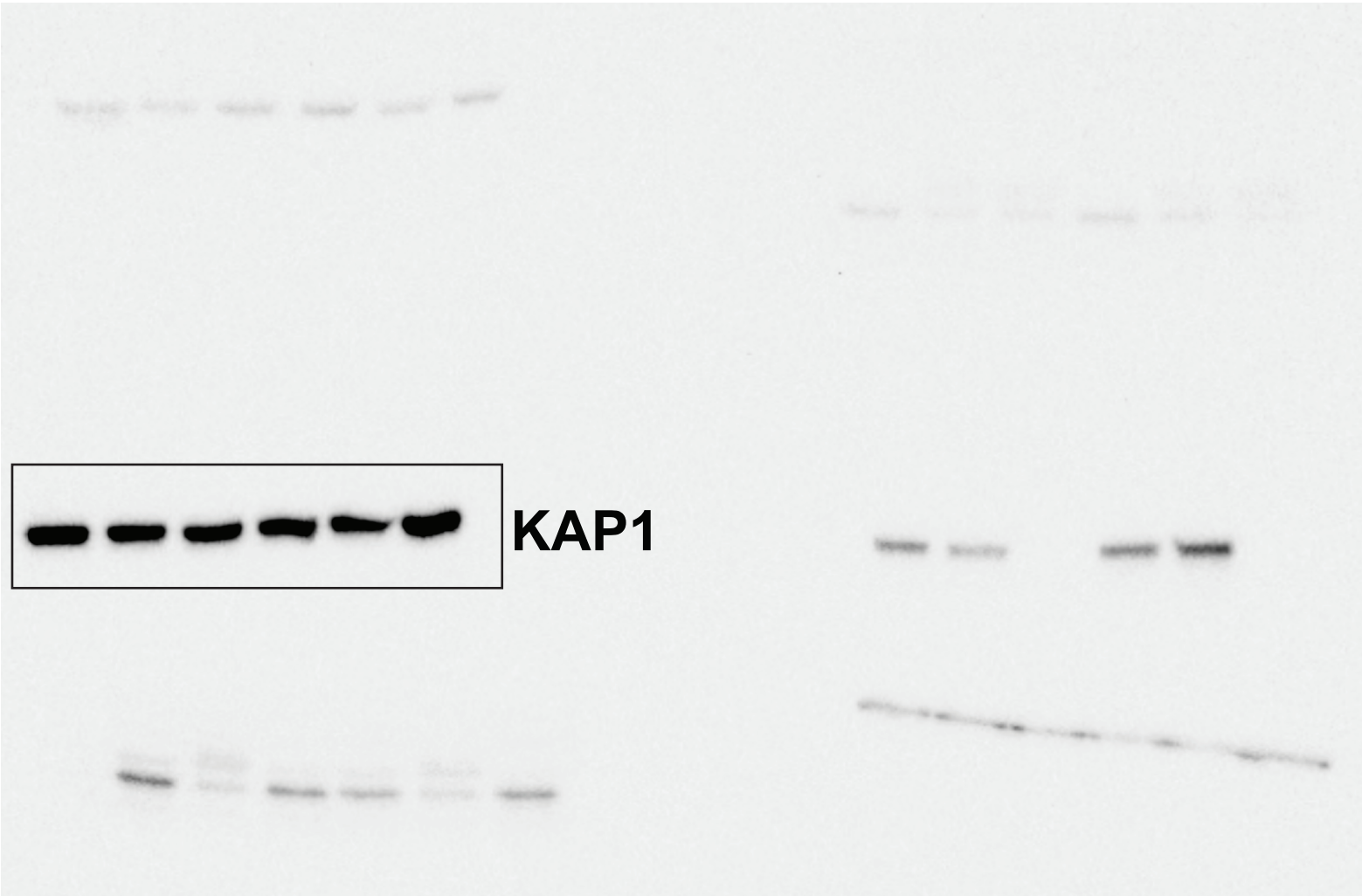

Supplement: Figure 2—source data 1. [file elife-90887-fig2-data1.zip › Figure 2_source_data/Figure_2_source_4.pdf]

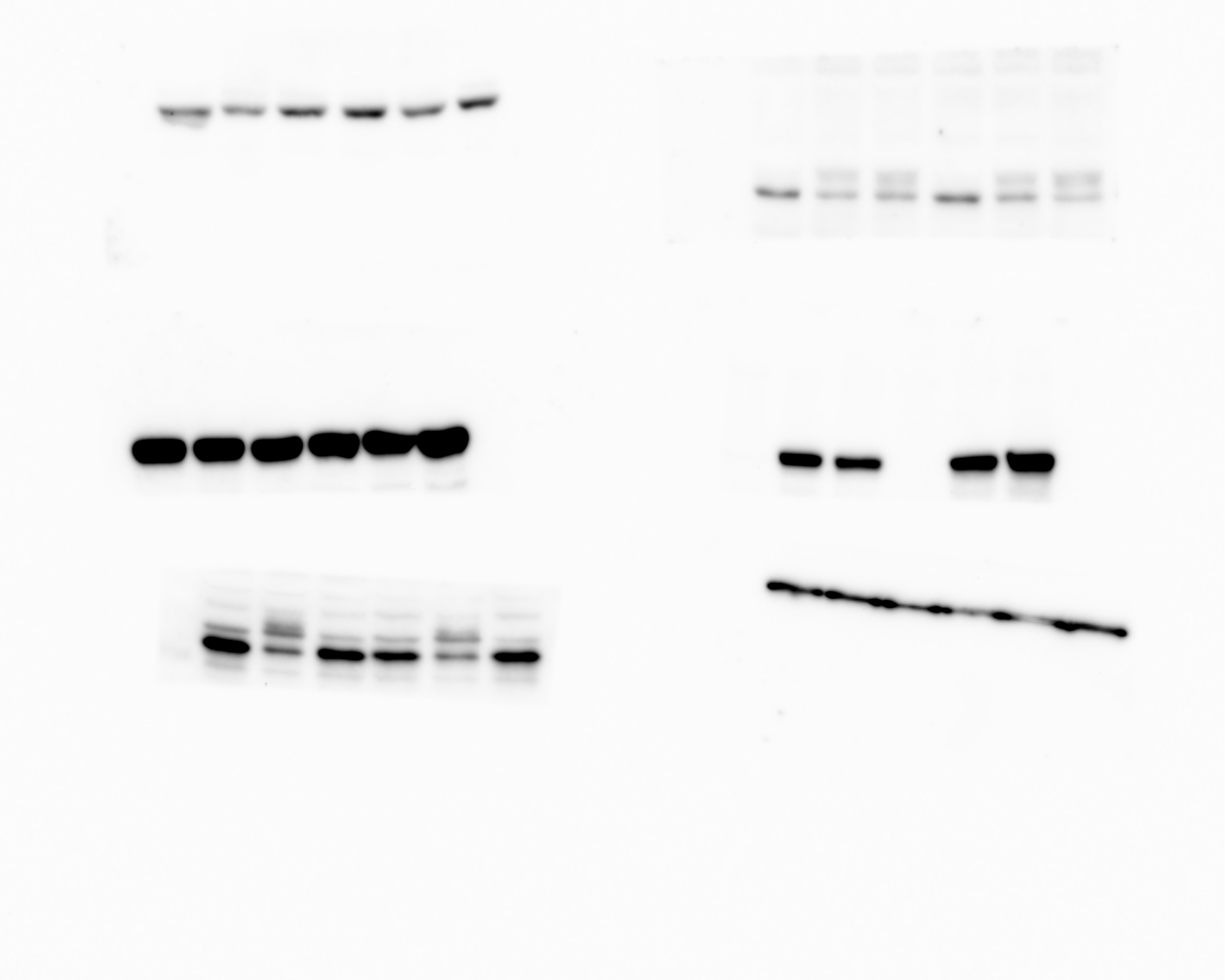

Supplement: Figure 2—source data 1. [file elife-90887-fig2-data1.zip › Figure 2_source_data/Figure_2_source_5.tif]

Figure 2 Source Data 6

E

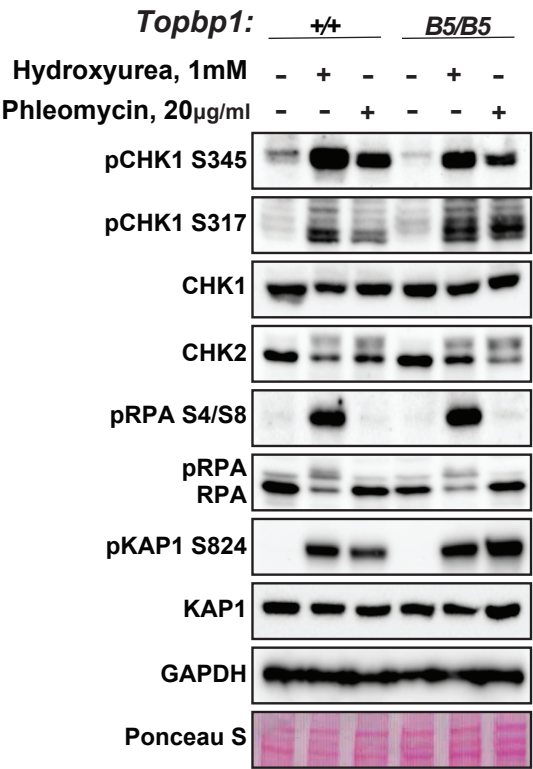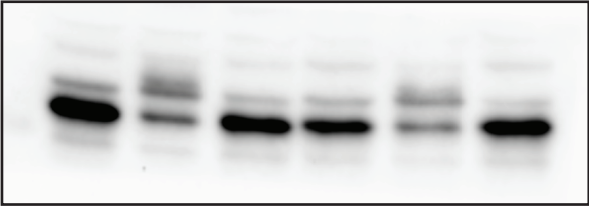

pRPA  
RPA

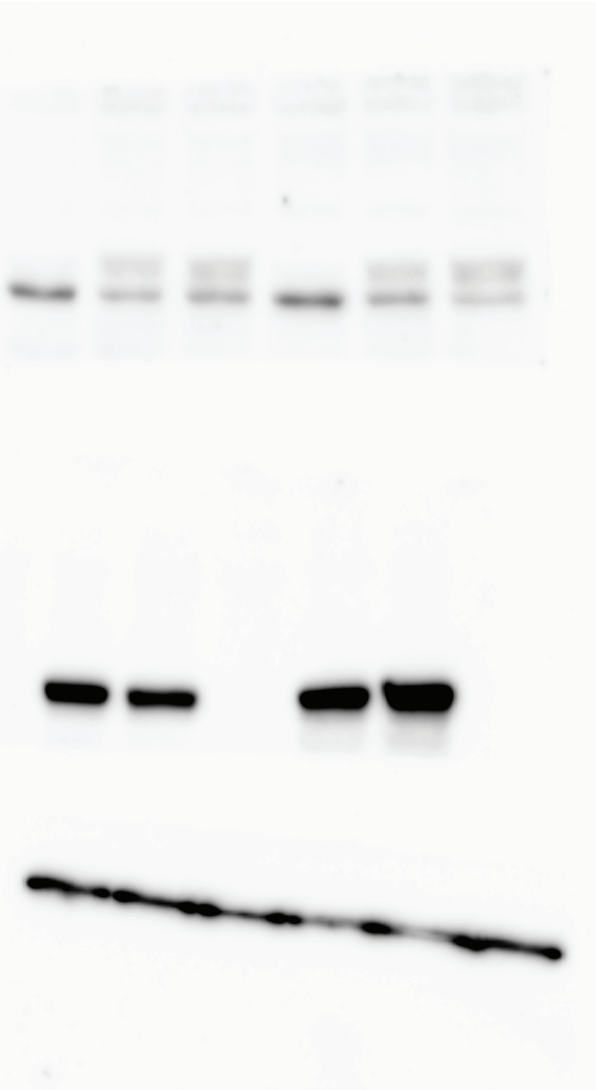

Supplement: Figure 2—source data 1. [file elife-90887-fig2-data1.zip › Figure 2_source_data/Figure_2_source_6.pdf]

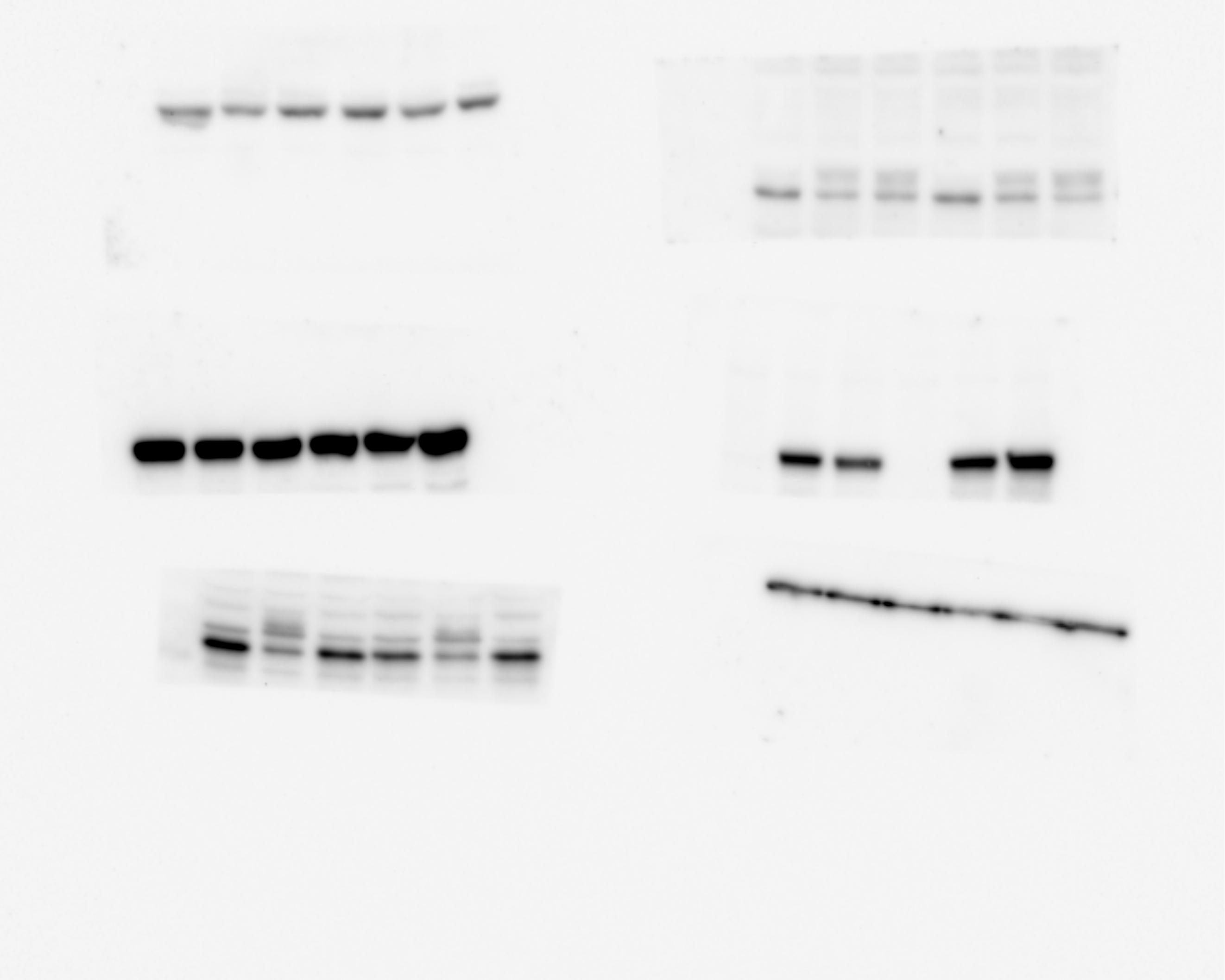

Supplement: Figure 2—source data 1. [file elife-90887-fig2-data1.zip › Figure 2_source_data/Figure_2_source_7.tif]

Figure 2 Source Data 8

E

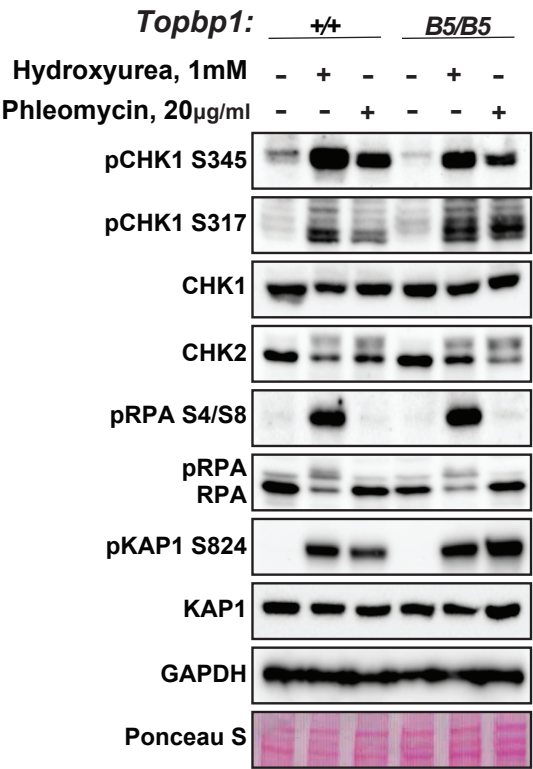

pKAP1 S824

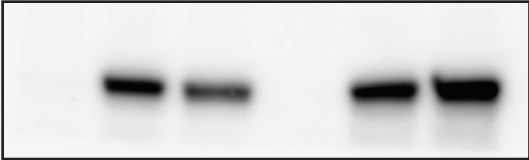

Supplement: Figure 2—source data 1. [file elife-90887-fig2-data1.zip › Figure 2_source_data/Figure_2_source_8.pdf]

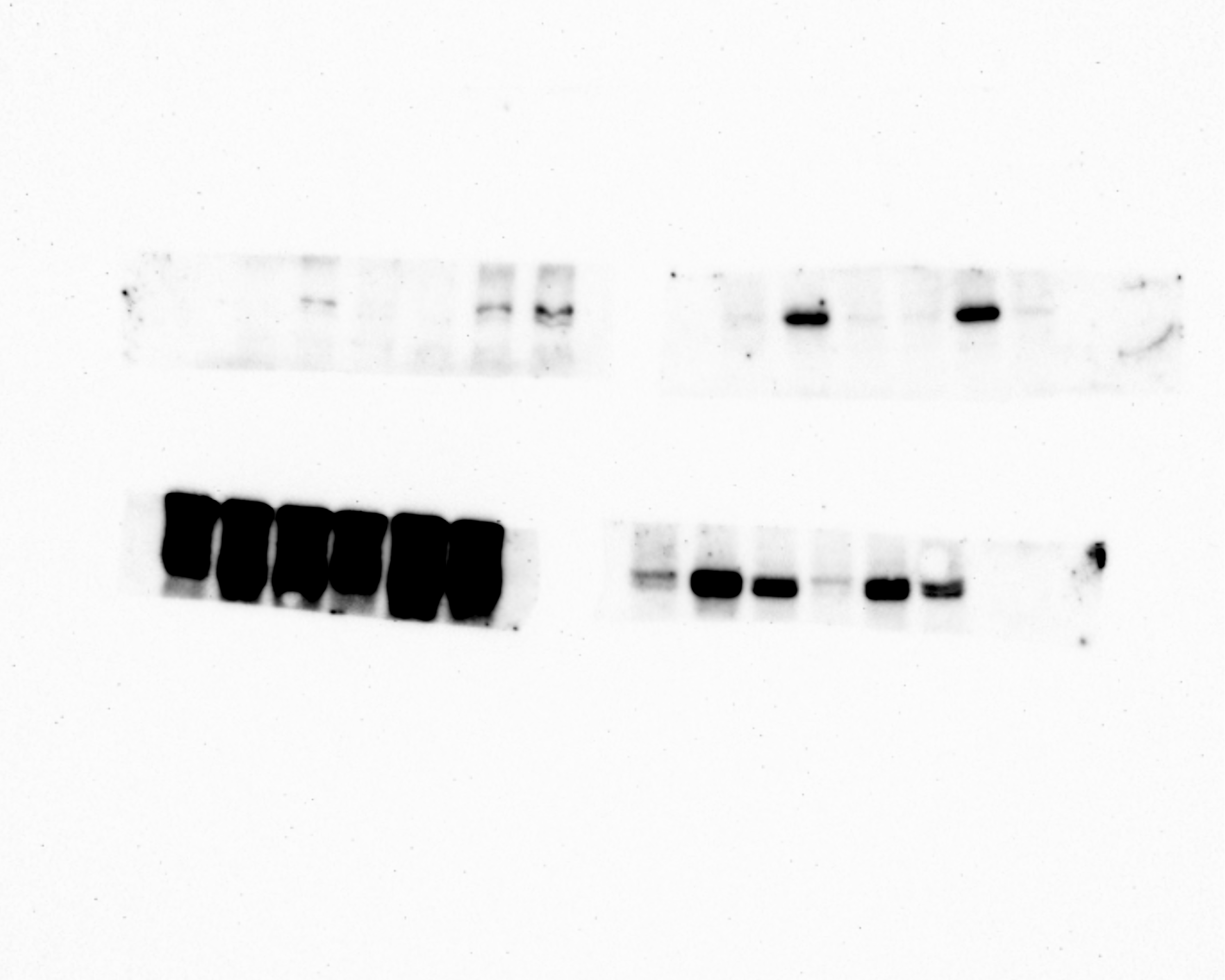

Supplement: Figure 2—source data 1. [file elife-90887-fig2-data1.zip › Figure 2_source_data/Figure_2_source_9.tif]

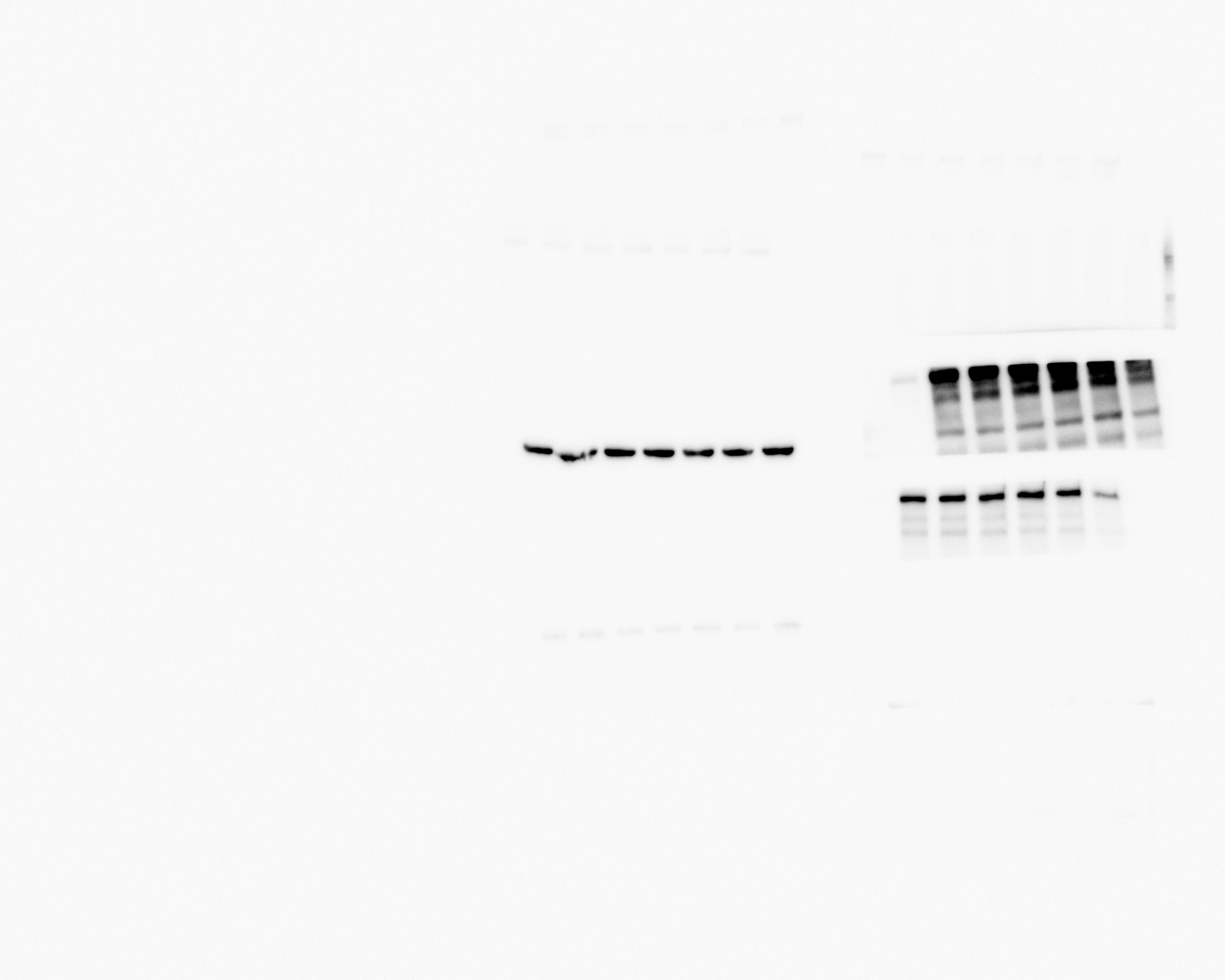

Supplement: Figure 2—figure supplement 3—source data 1. [file elife-90887-fig2-figsupp3-data1.zip › Figure 2-figure supplement_3_souece_data/Figure 2-figure supplement 3_source_data_1.tif]

Figure 2-figure supplement 3\_source\_data\_10

A

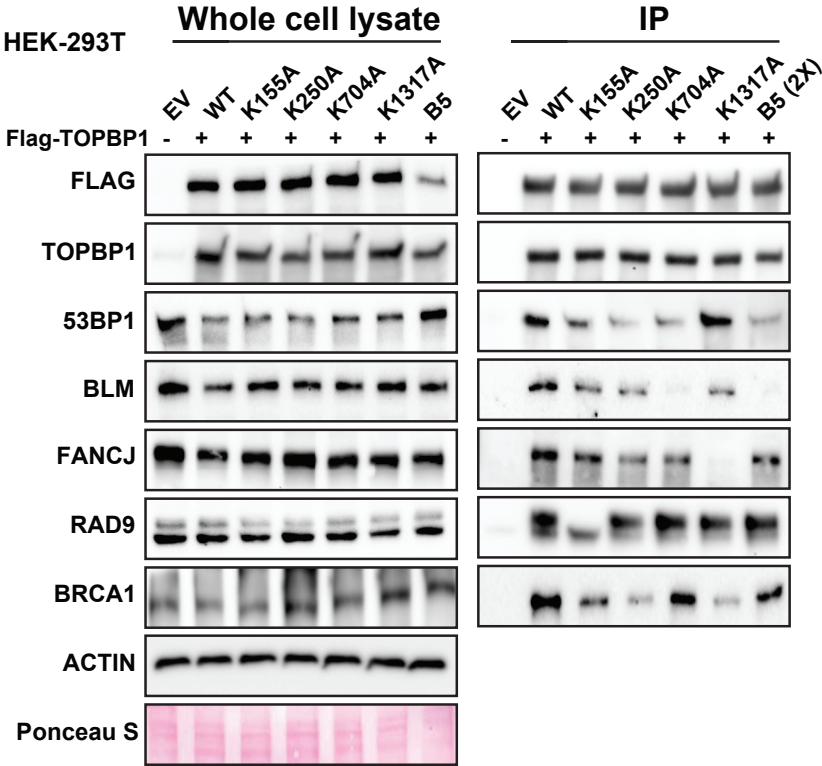

Whole cell lysate

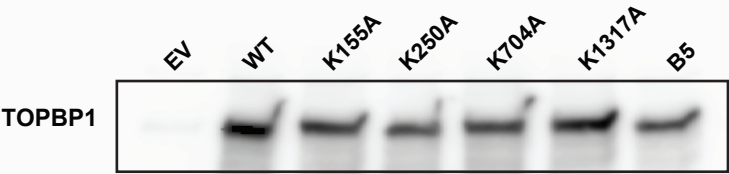

Supplement: Figure 2—figure supplement 3—source data 1. [file elife-90887-fig2-figsupp3-data1.zip › Figure 2-figure supplement_3_souece_data/Figure 2-figure supplement 3_source_data_10.pdf]

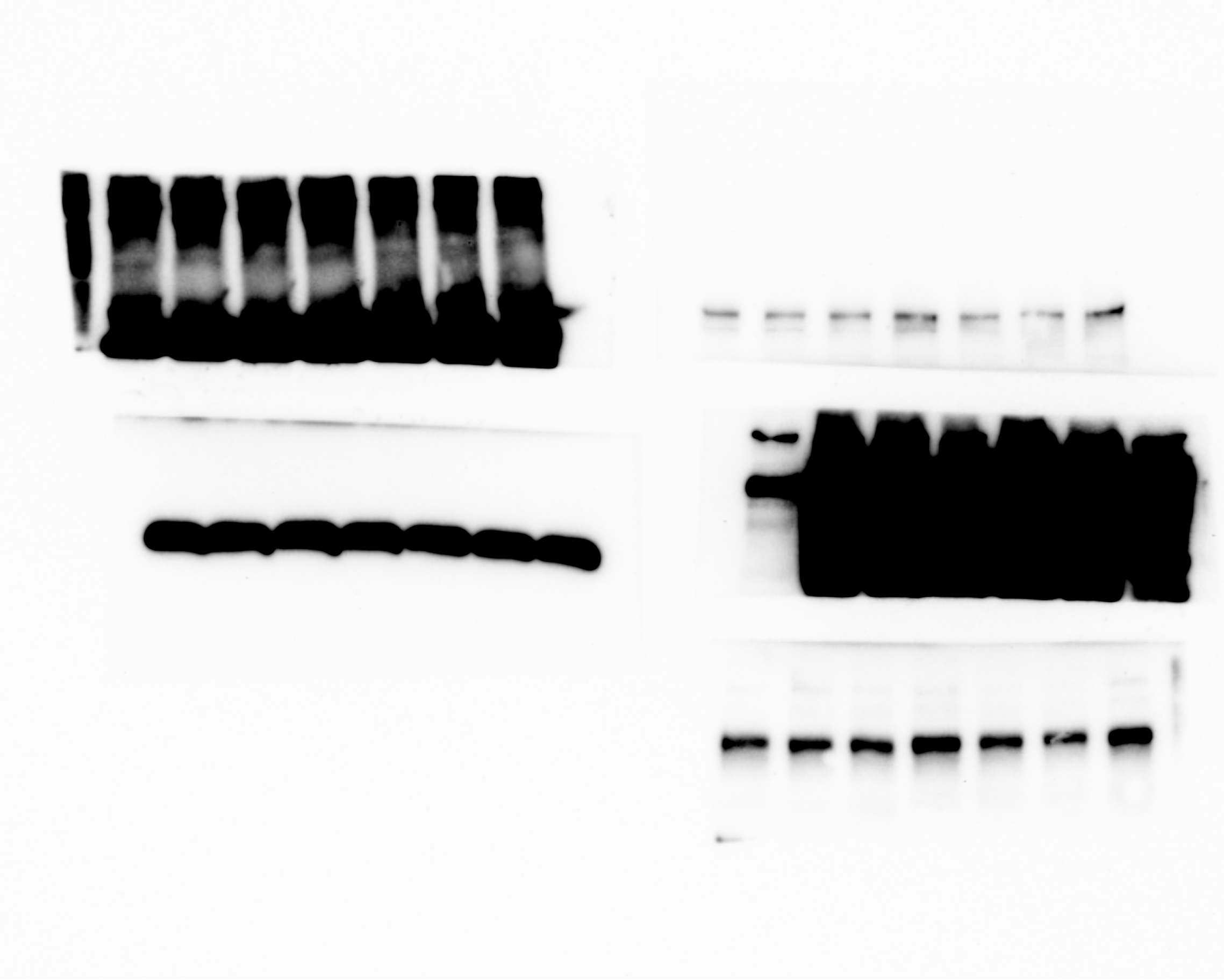

Supplement: Figure 2—figure supplement 3—source data 1. [file elife-90887-fig2-figsupp3-data1.zip › Figure 2-figure supplement_3_souece_data/Figure 2-figure supplement 3_source_data_11.tif]

Figure 2-figure supplement 3\_source\_data\_12

A

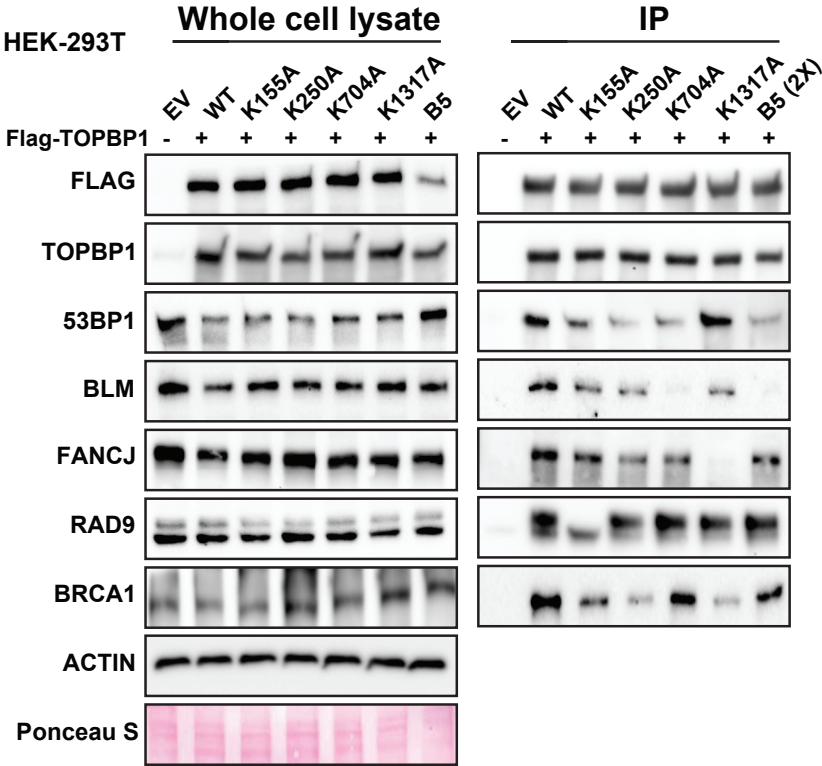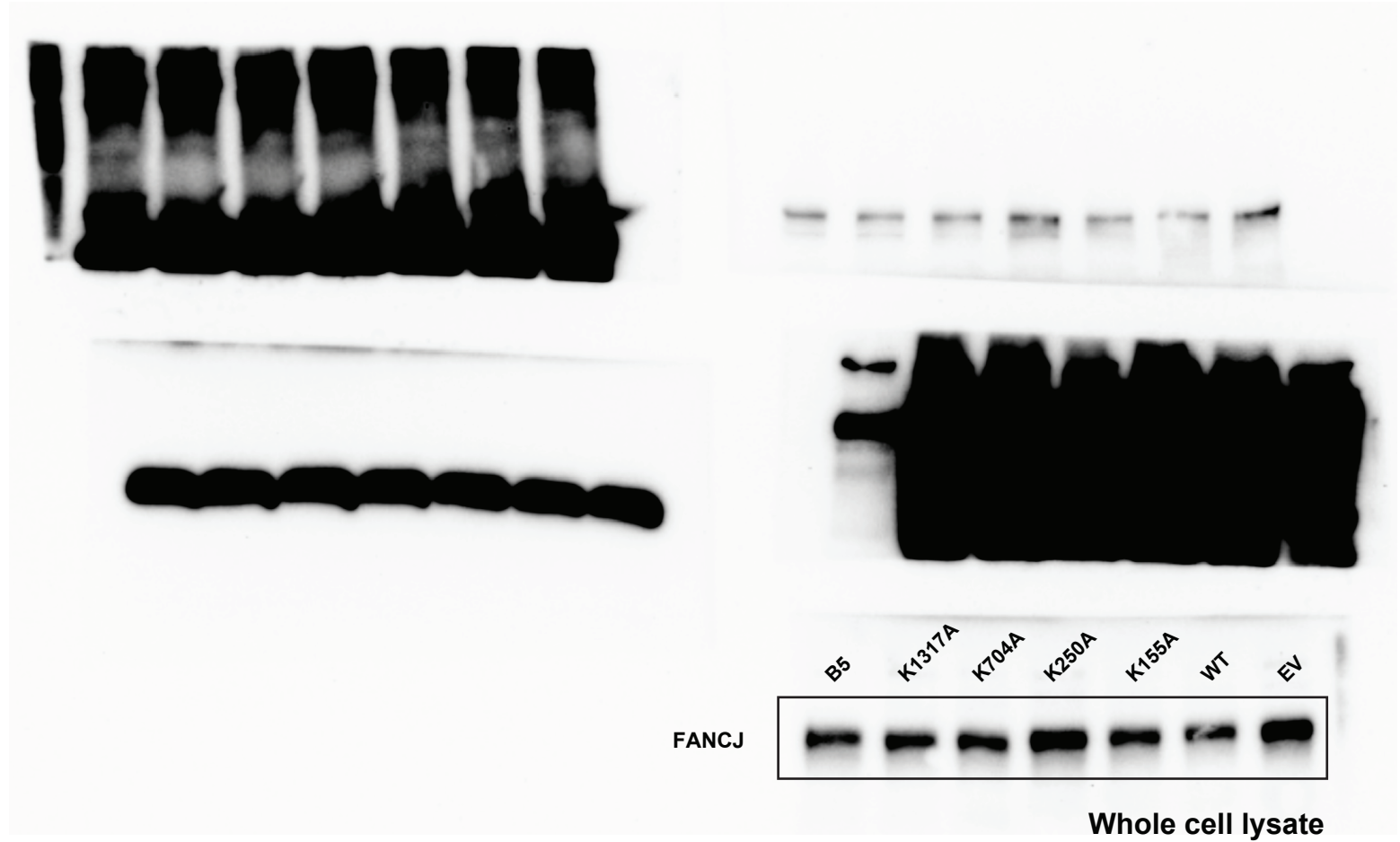

Supplement: Figure 2—figure supplement 3—source data 1. [file elife-90887-fig2-figsupp3-data1.zip › Figure 2-figure supplement_3_souece_data/Figure 2-figure supplement 3_source_data_12.pdf]

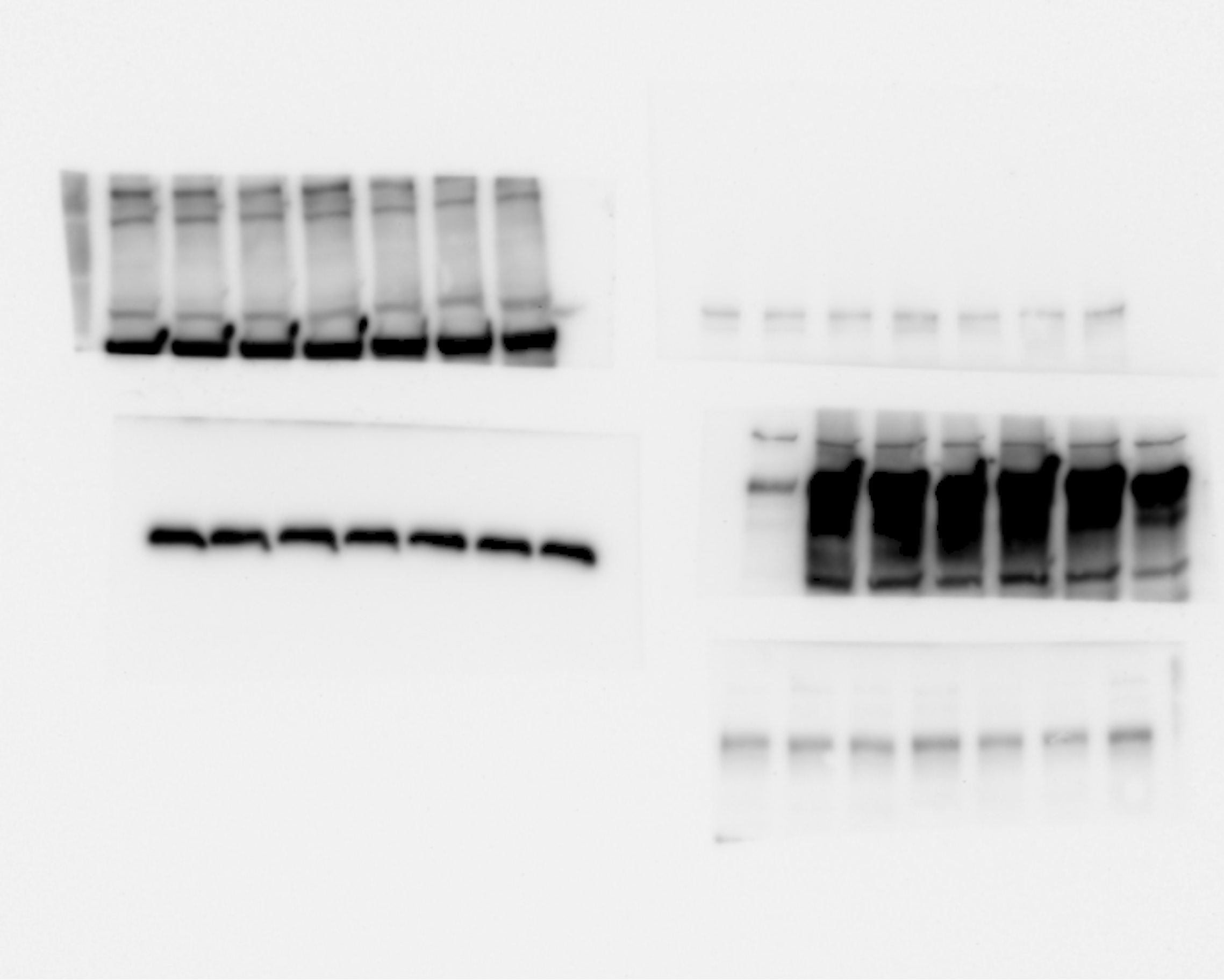

Supplement: Figure 2—figure supplement 3—source data 1. [file elife-90887-fig2-figsupp3-data1.zip › Figure 2-figure supplement_3_souece_data/Figure 2-figure supplement 3_source_data_13.tif]

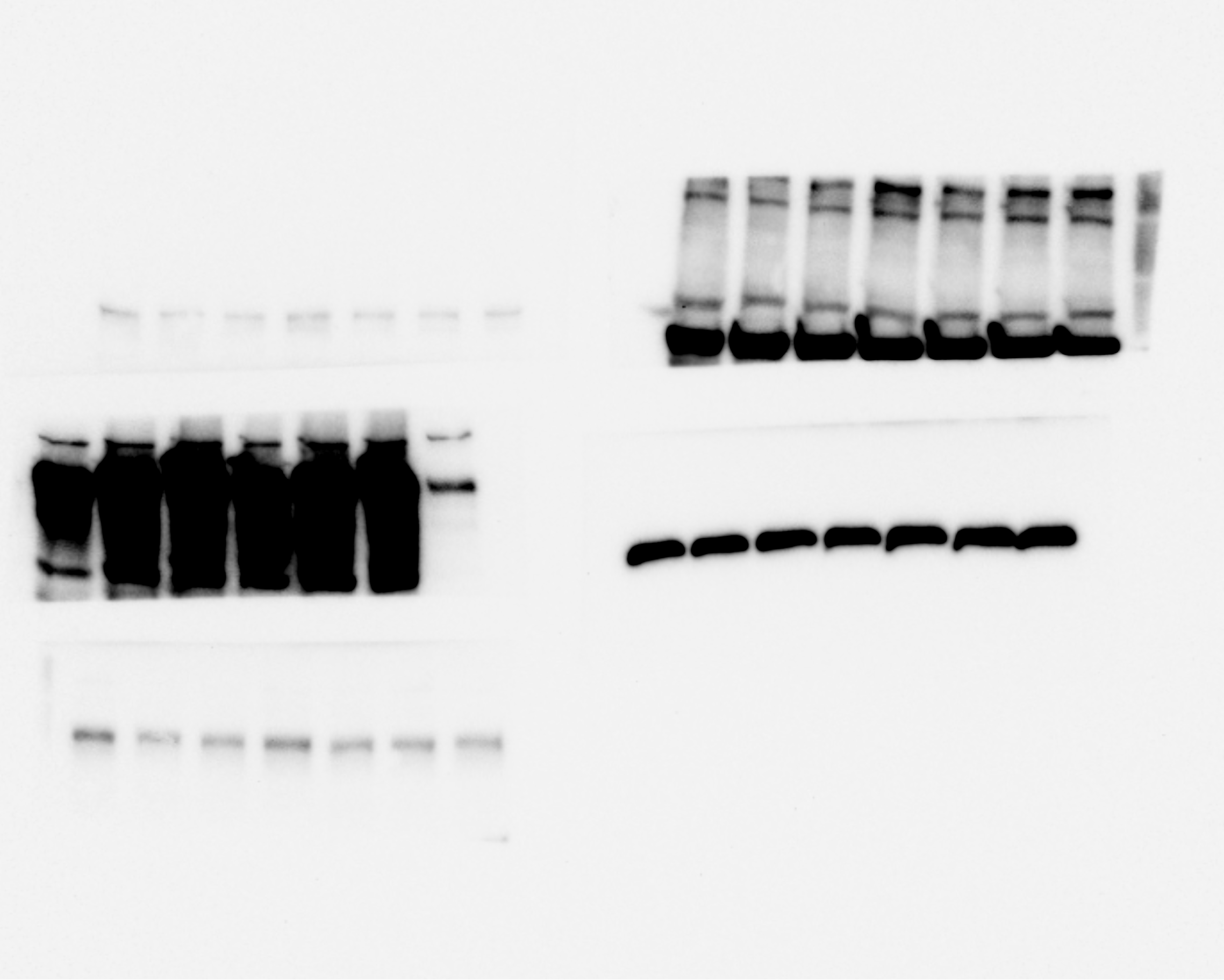

Supplement: Figure 2—figure supplement 3—source data 1. [file elife-90887-fig2-figsupp3-data1.zip › Figure 2-figure supplement_3_souece_data/Figure 2-figure supplement 3_source_data_15.tif]

Figure 2-figure supplement 3\_source\_data\_16

A

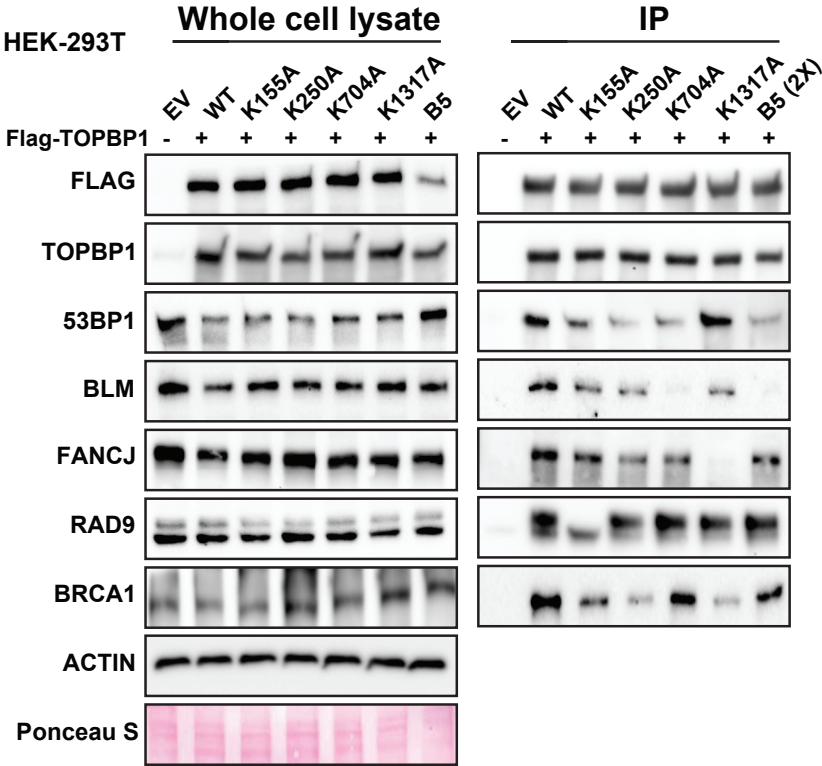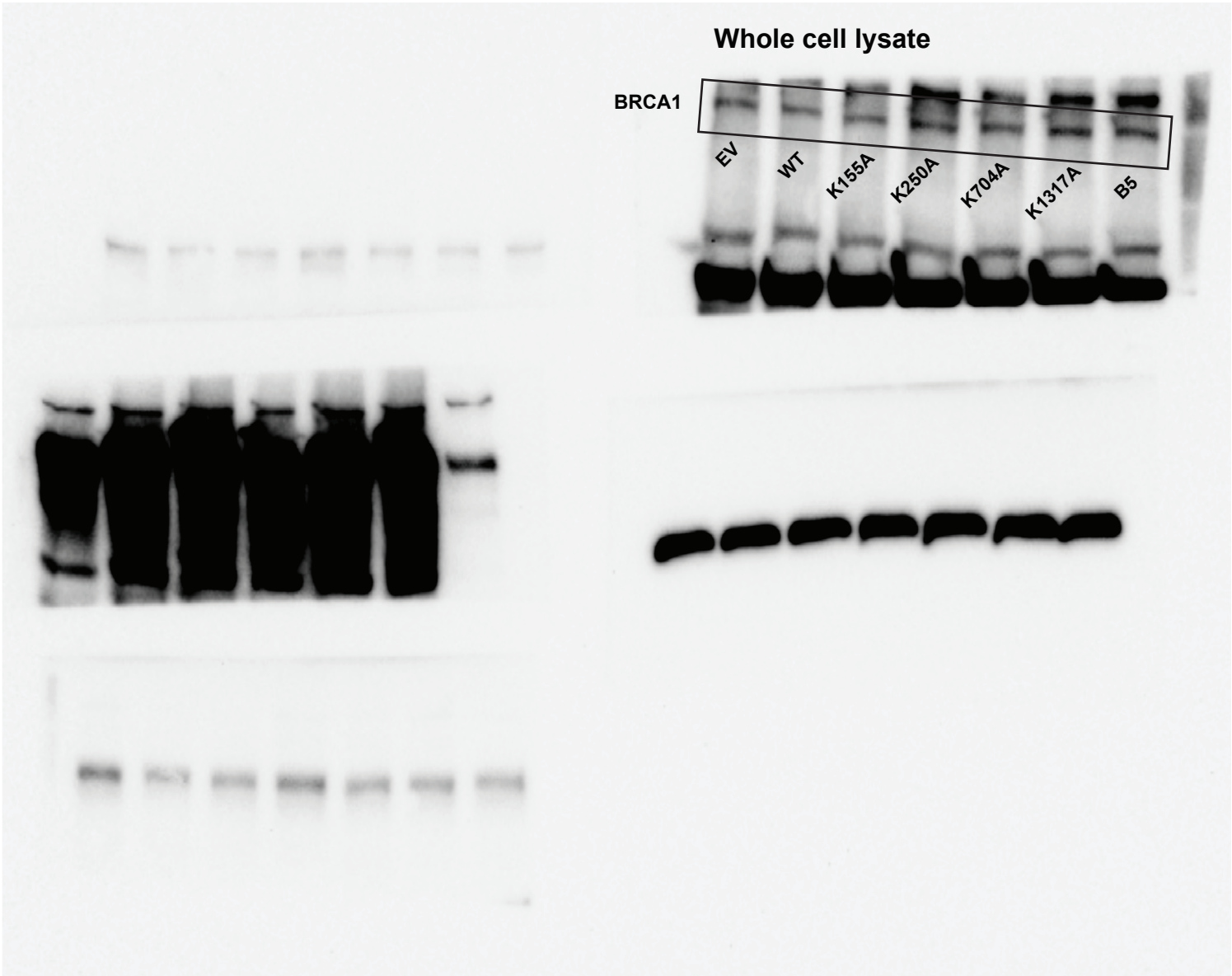

Supplement: Figure 2—figure supplement 3—source data 1. [file elife-90887-fig2-figsupp3-data1.zip › Figure 2-figure supplement_3_souece_data/Figure 2-figure supplement 3_source_data_16.pdf]

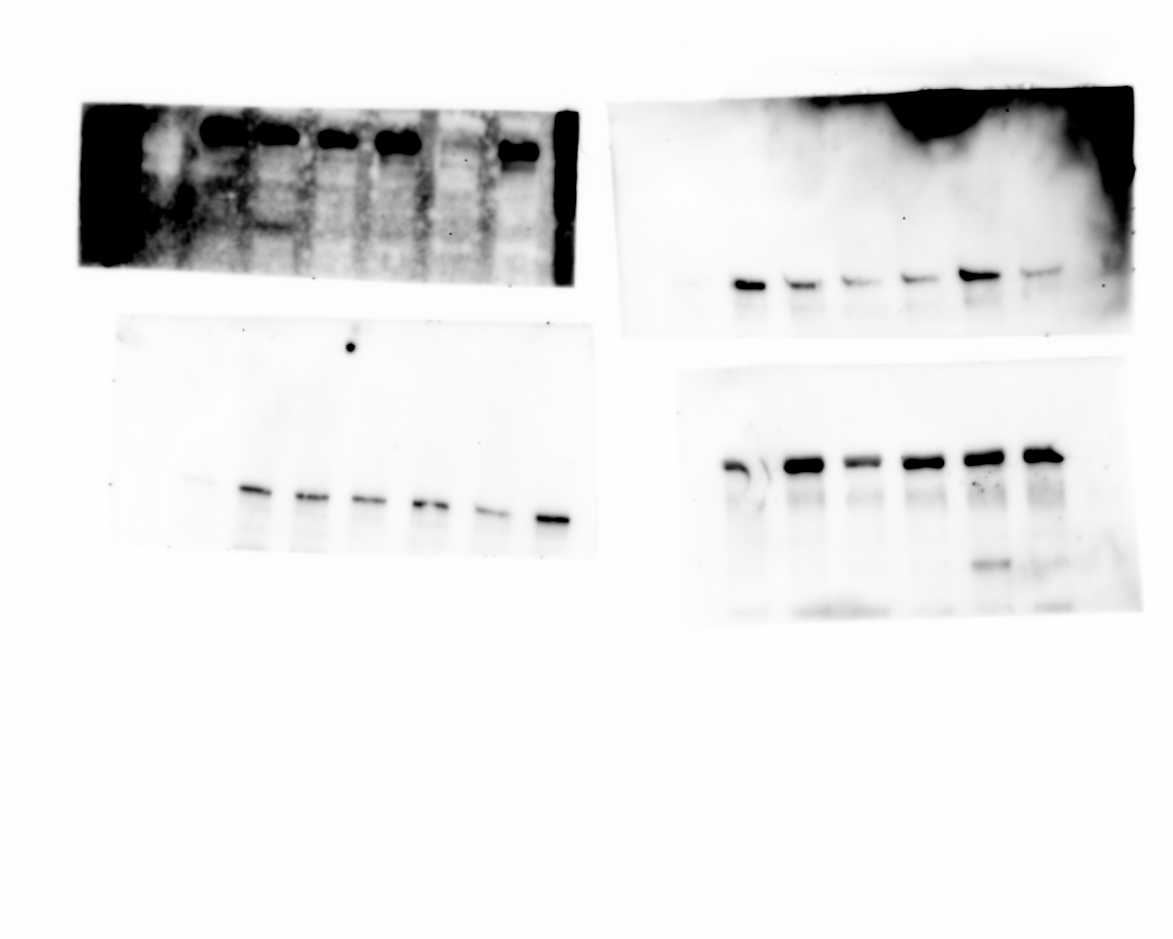

Supplement: Figure 2—figure supplement 3—source data 1. [file elife-90887-fig2-figsupp3-data1.zip › Figure 2-figure supplement_3_souece_data/Figure 2-figure supplement 3_source_data_17.tif]

Figure 2-figure supplement 3\_source\_data\_18

A

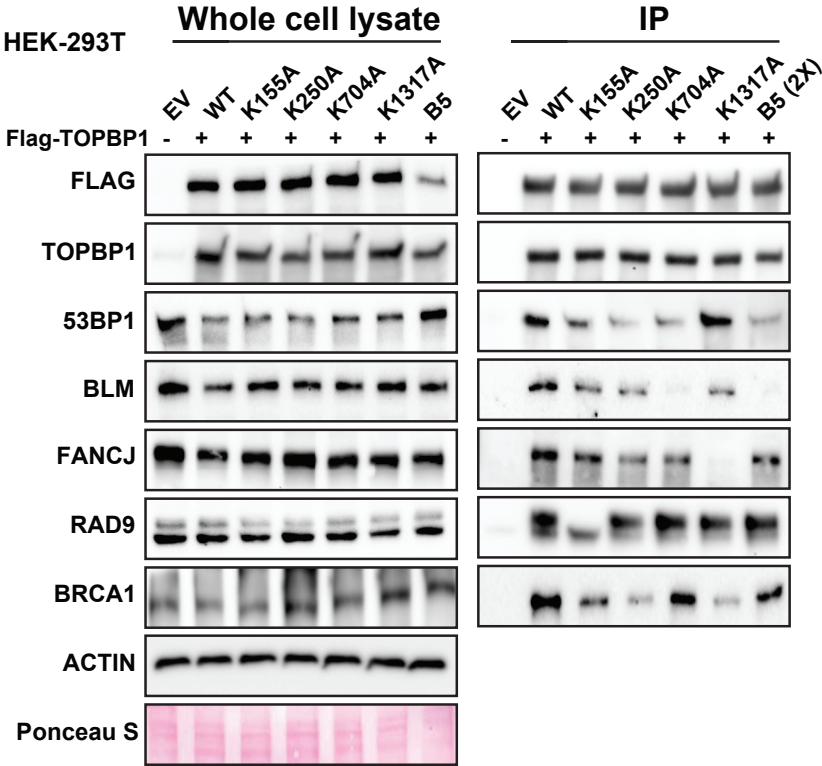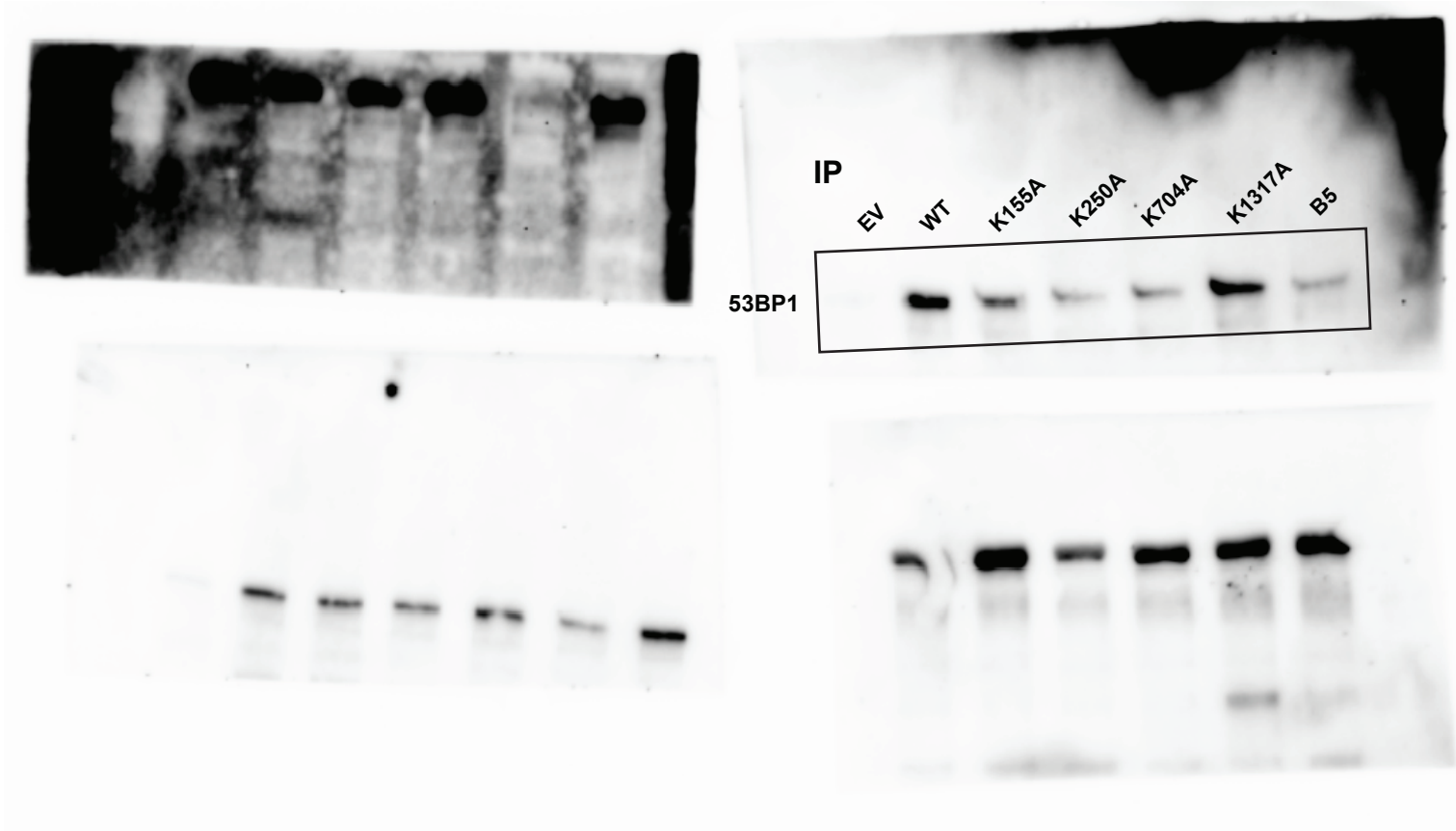

Supplement: Figure 2—figure supplement 3—source data 1. [file elife-90887-fig2-figsupp3-data1.zip › Figure 2-figure supplement_3_souece_data/Figure 2-figure supplement 3_source_data_18.pdf]

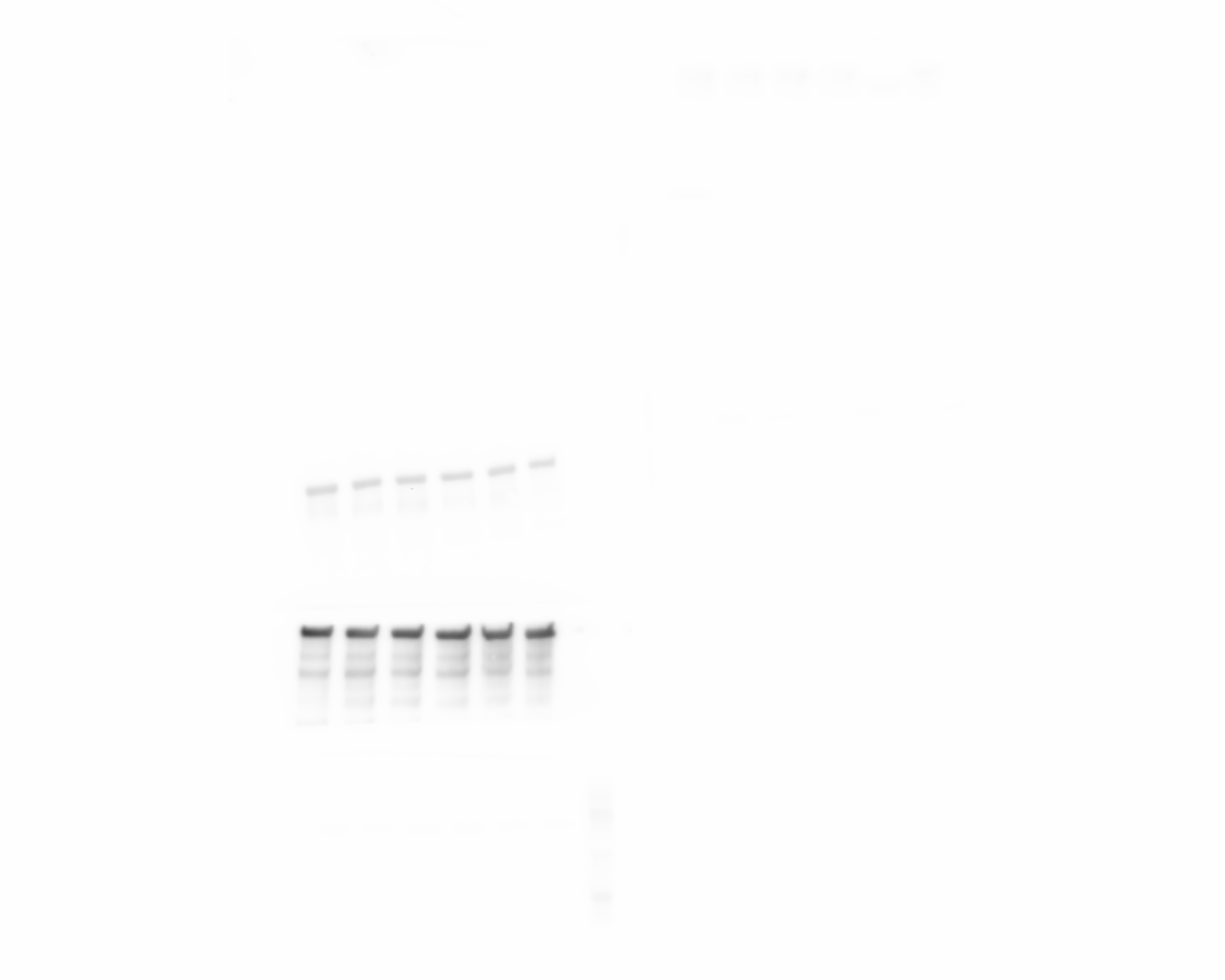

Supplement: Figure 2—figure supplement 3—source data 1. [file elife-90887-fig2-figsupp3-data1.zip › Figure 2-figure supplement_3_souece_data/Figure 2-figure supplement 3_source_data_19.tif]

Figure 2-figure supplement 3\_source\_data\_2

A

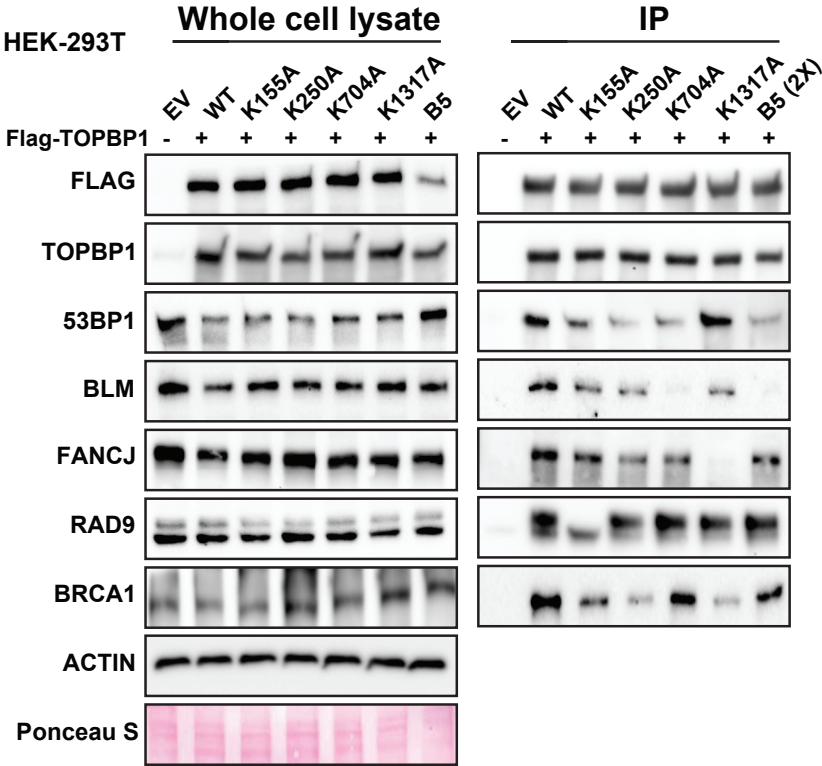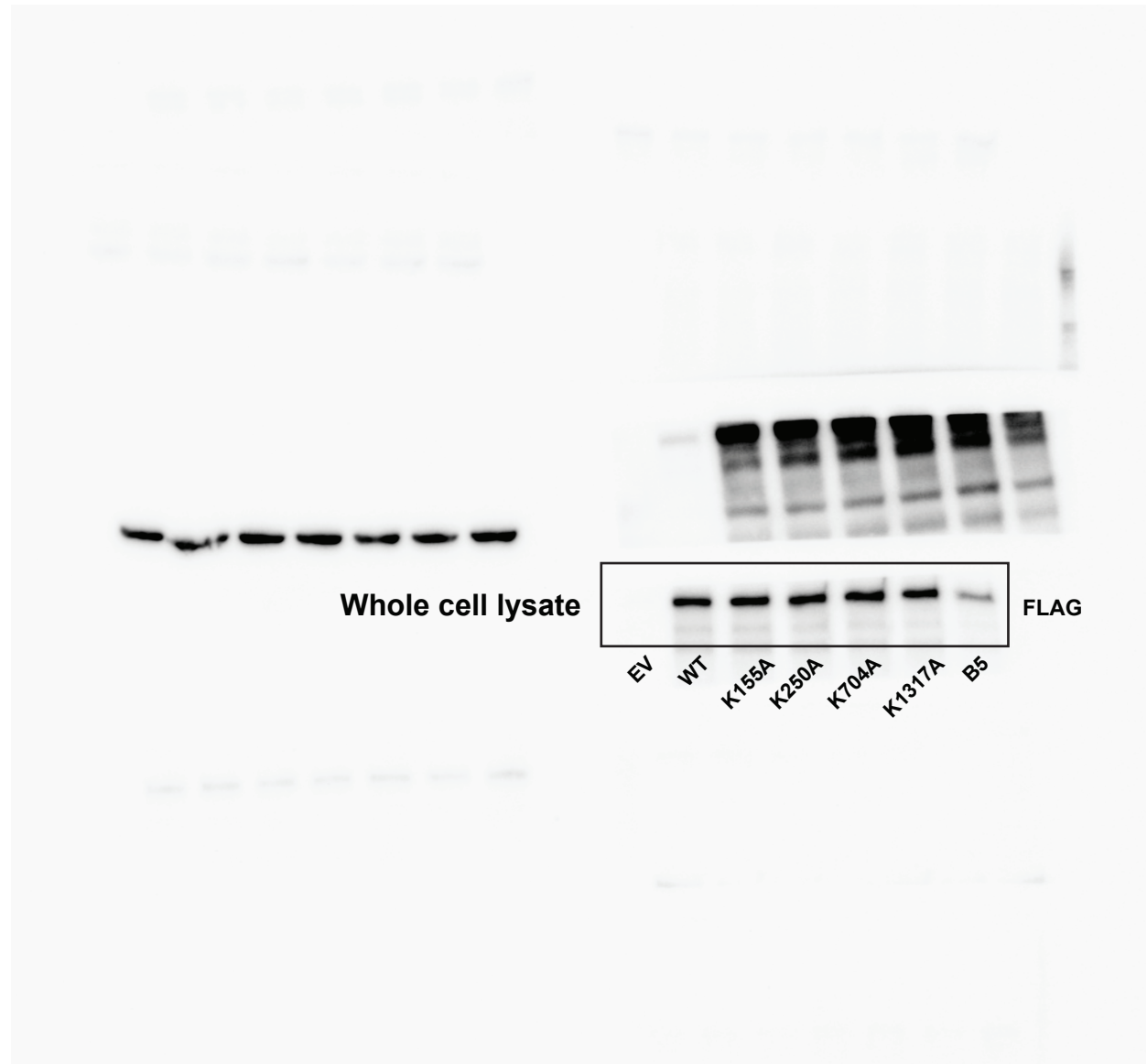

Supplement: Figure 2—figure supplement 3—source data 1. [file elife-90887-fig2-figsupp3-data1.zip › Figure 2-figure supplement_3_souece_data/Figure 2-figure supplement 3_source_data_2.pdf]

Figure 2-figure supplement 3\_source\_data\_20

A

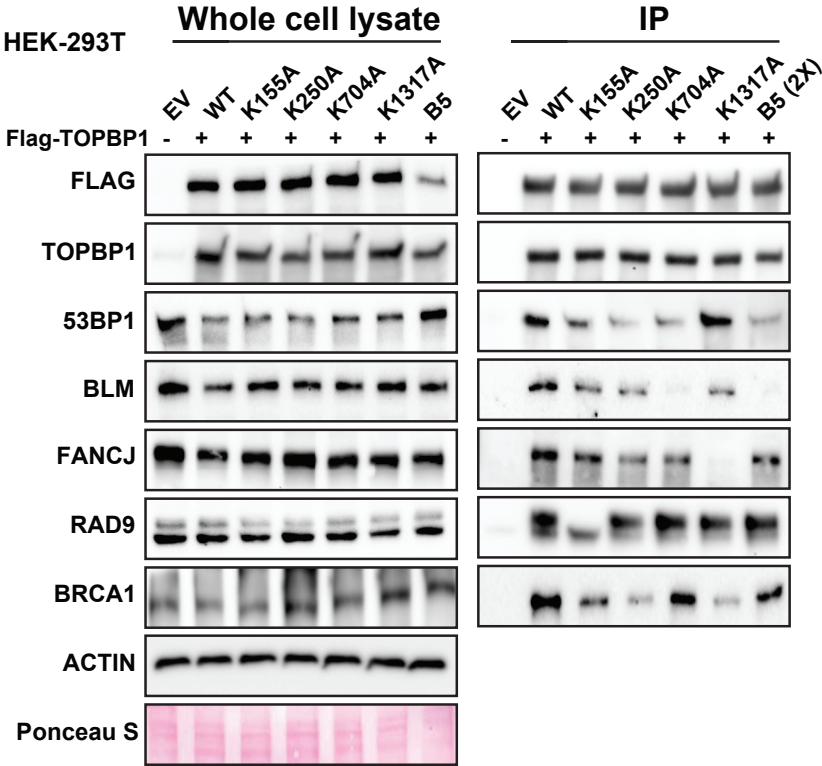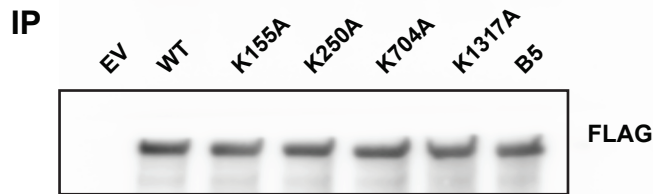

Supplement: Figure 2—figure supplement 3—source data 1. [file elife-90887-fig2-figsupp3-data1.zip › Figure 2-figure supplement_3_souece_data/Figure 2-figure supplement 3_source_data_20.pdf]

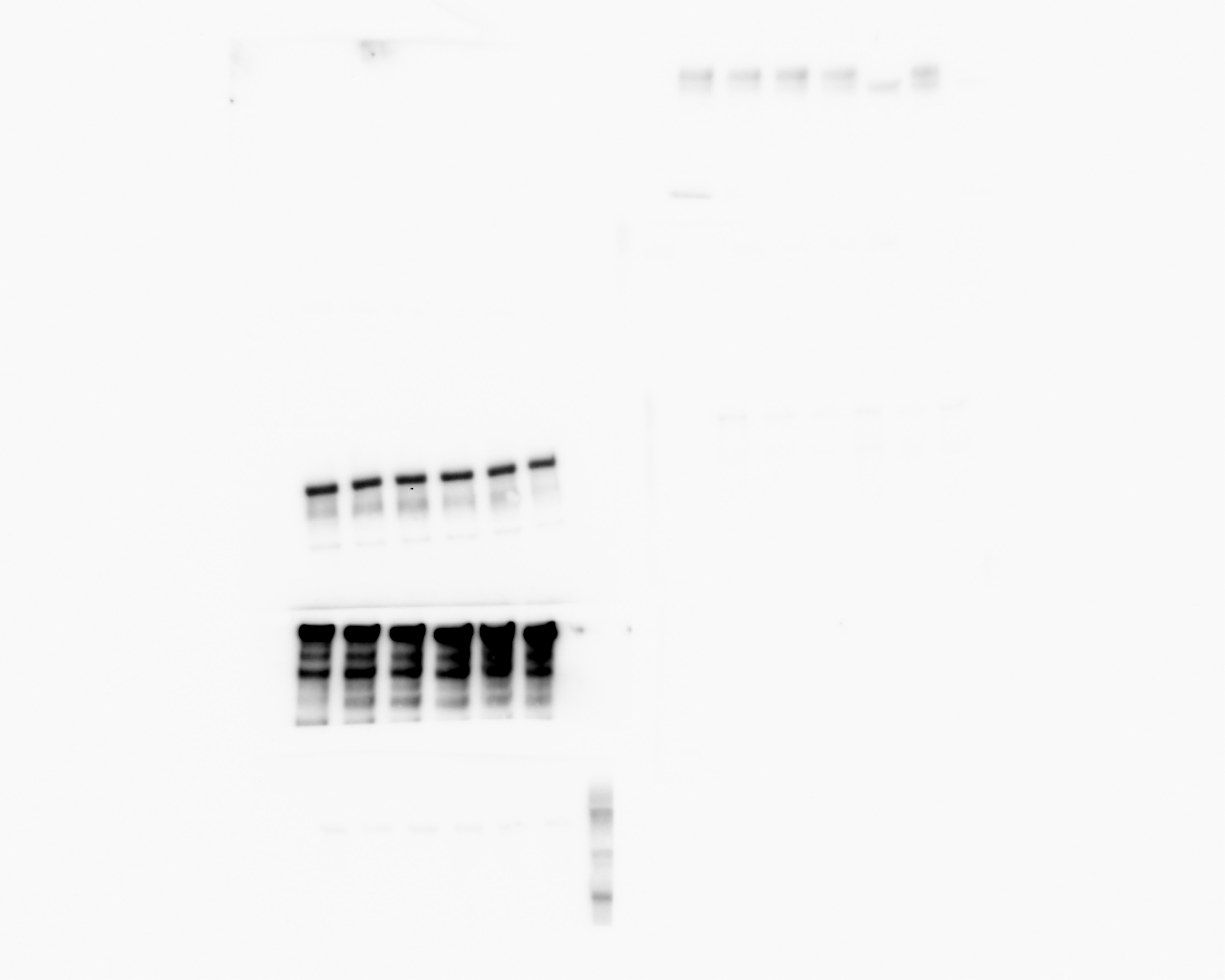

Supplement: Figure 2—figure supplement 3—source data 1. [file elife-90887-fig2-figsupp3-data1.zip › Figure 2-figure supplement_3_souece_data/Figure 2-figure supplement 3_source_data_21.tif]

Figure 2-figure supplement 3\_source\_data\_22

A

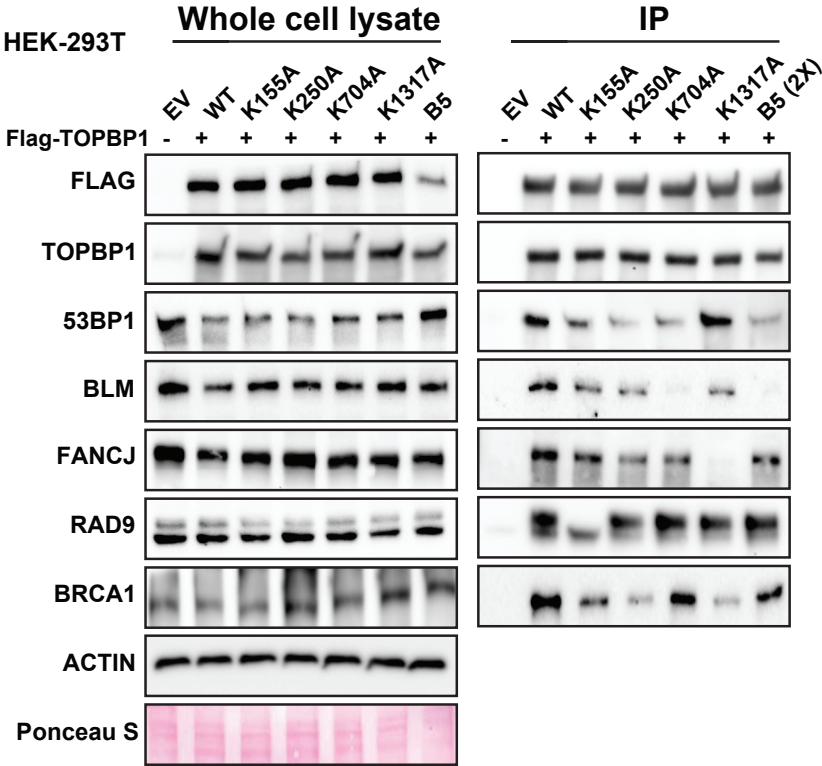

IP

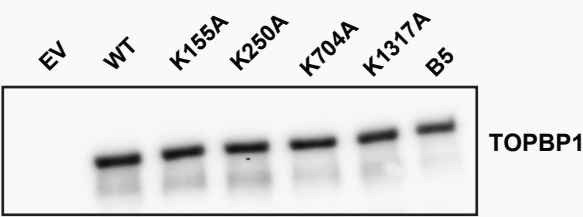

Supplement: Figure 2—figure supplement 3—source data 1. [file elife-90887-fig2-figsupp3-data1.zip › Figure 2-figure supplement_3_souece_data/Figure 2-figure supplement 3_source_data_22.pdf]

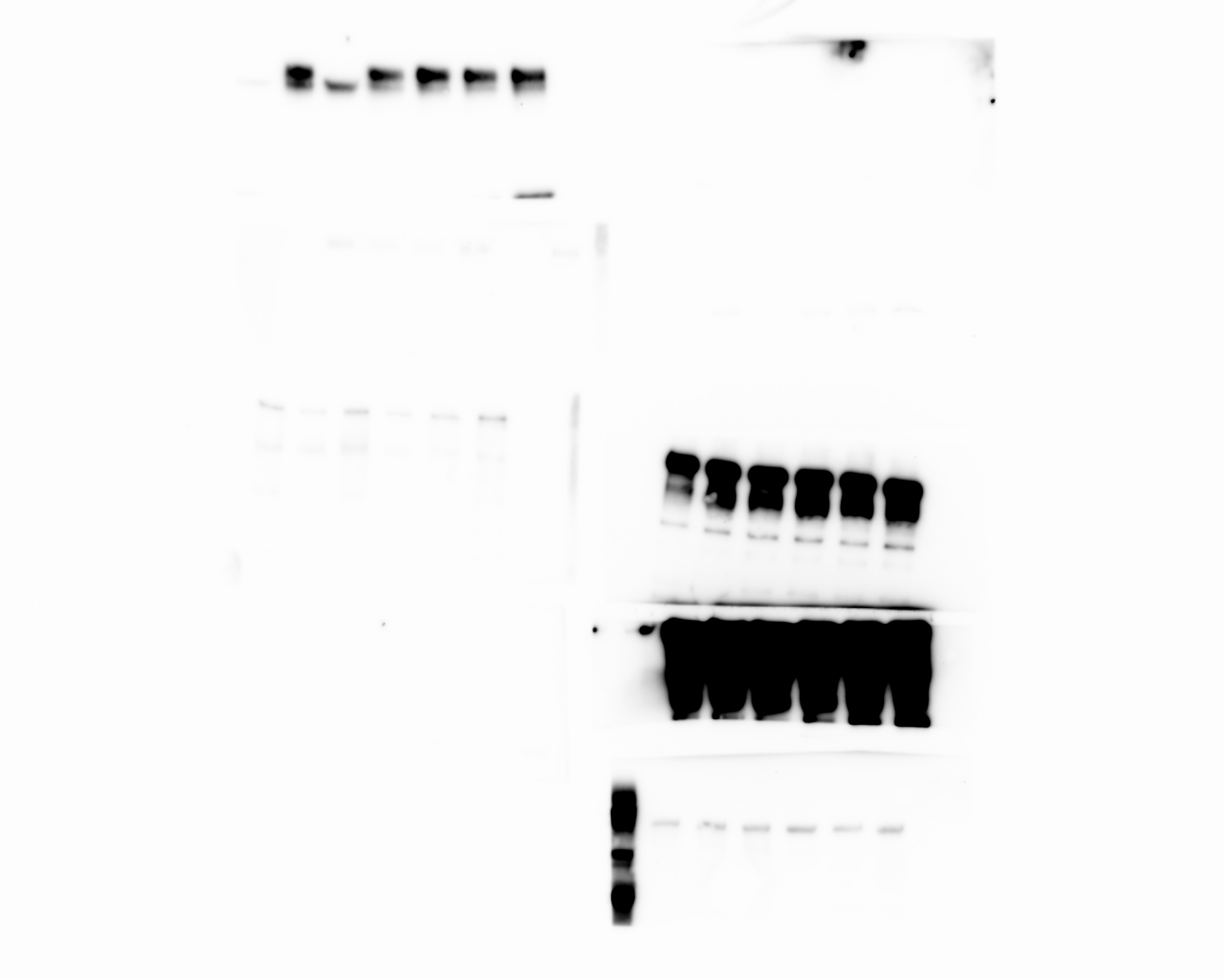

Supplement: Figure 2—figure supplement 3—source data 1. [file elife-90887-fig2-figsupp3-data1.zip › Figure 2-figure supplement_3_souece_data/Figure 2-figure supplement 3_source_data_23.tif]

Figure 2-figure supplement 3\_source\_data\_24

A

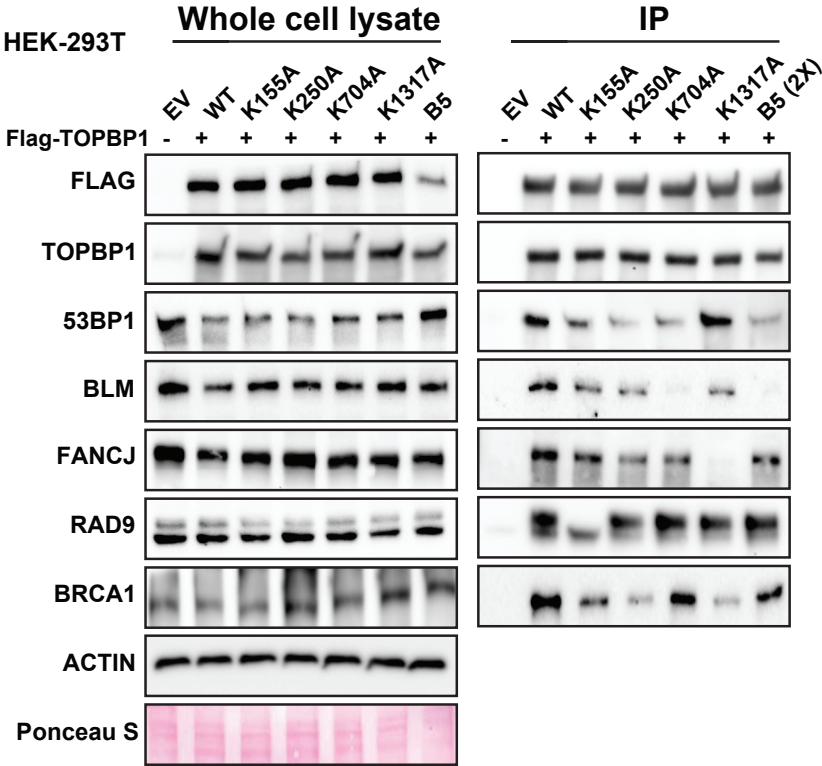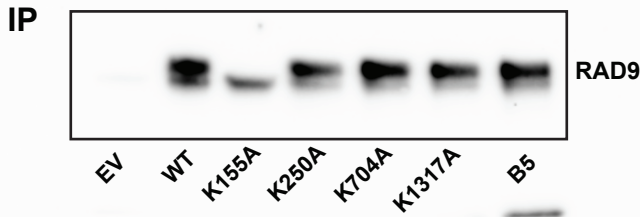

Supplement: Figure 2—figure supplement 3—source data 1. [file elife-90887-fig2-figsupp3-data1.zip › Figure 2-figure supplement_3_souece_data/Figure 2-figure supplement 3_source_data_24.pdf]

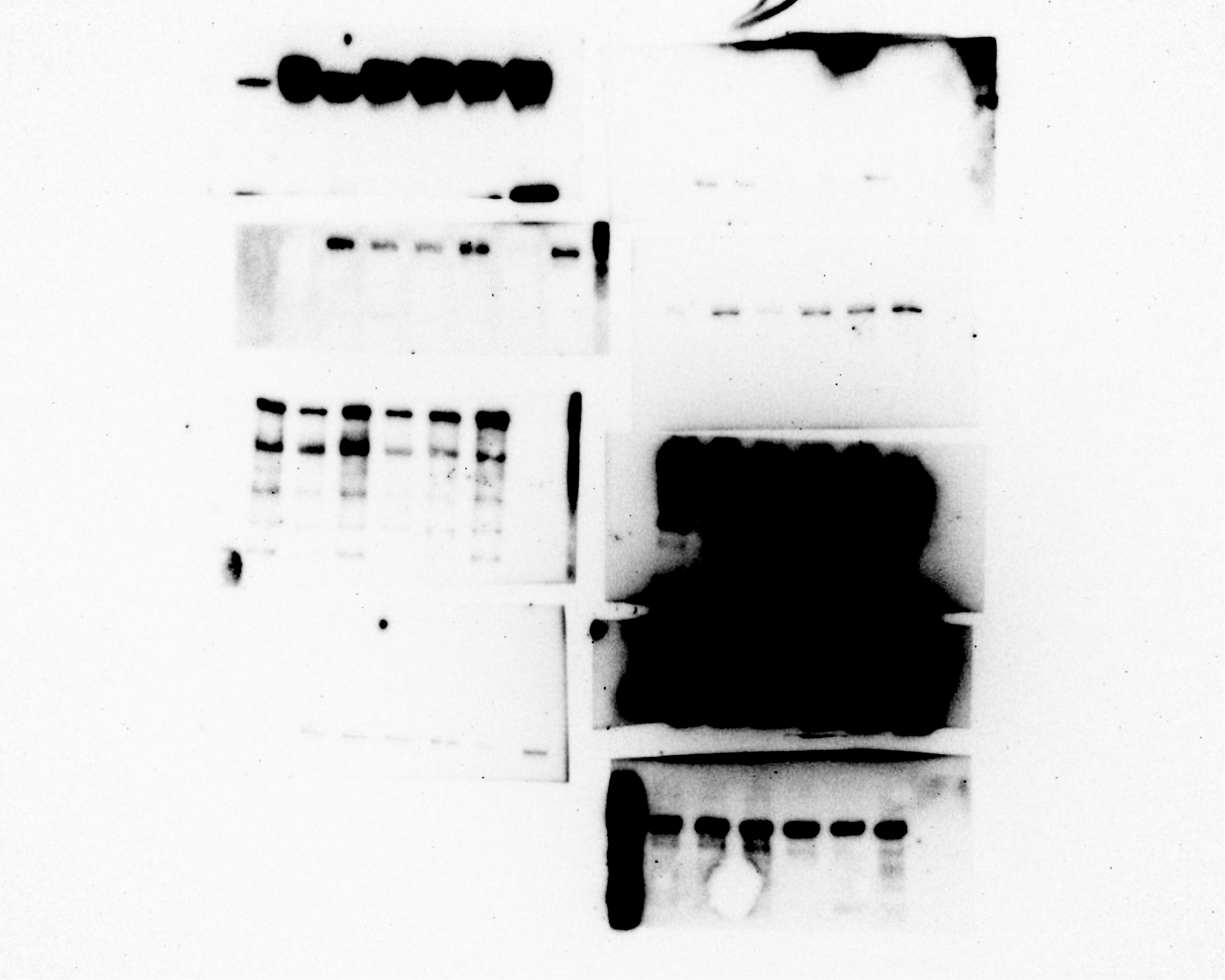

Supplement: Figure 2—figure supplement 3—source data 1. [file elife-90887-fig2-figsupp3-data1.zip › Figure 2-figure supplement_3_souece_data/Figure 2-figure supplement 3_source_data_25.tif]

Figure 2-figure supplement 3\_source\_data\_26

A

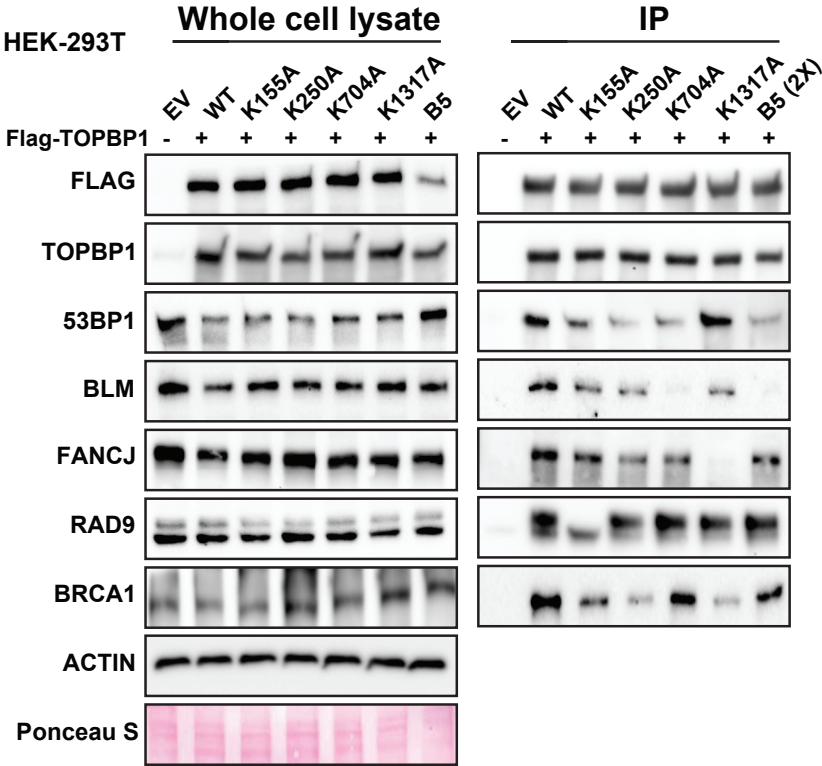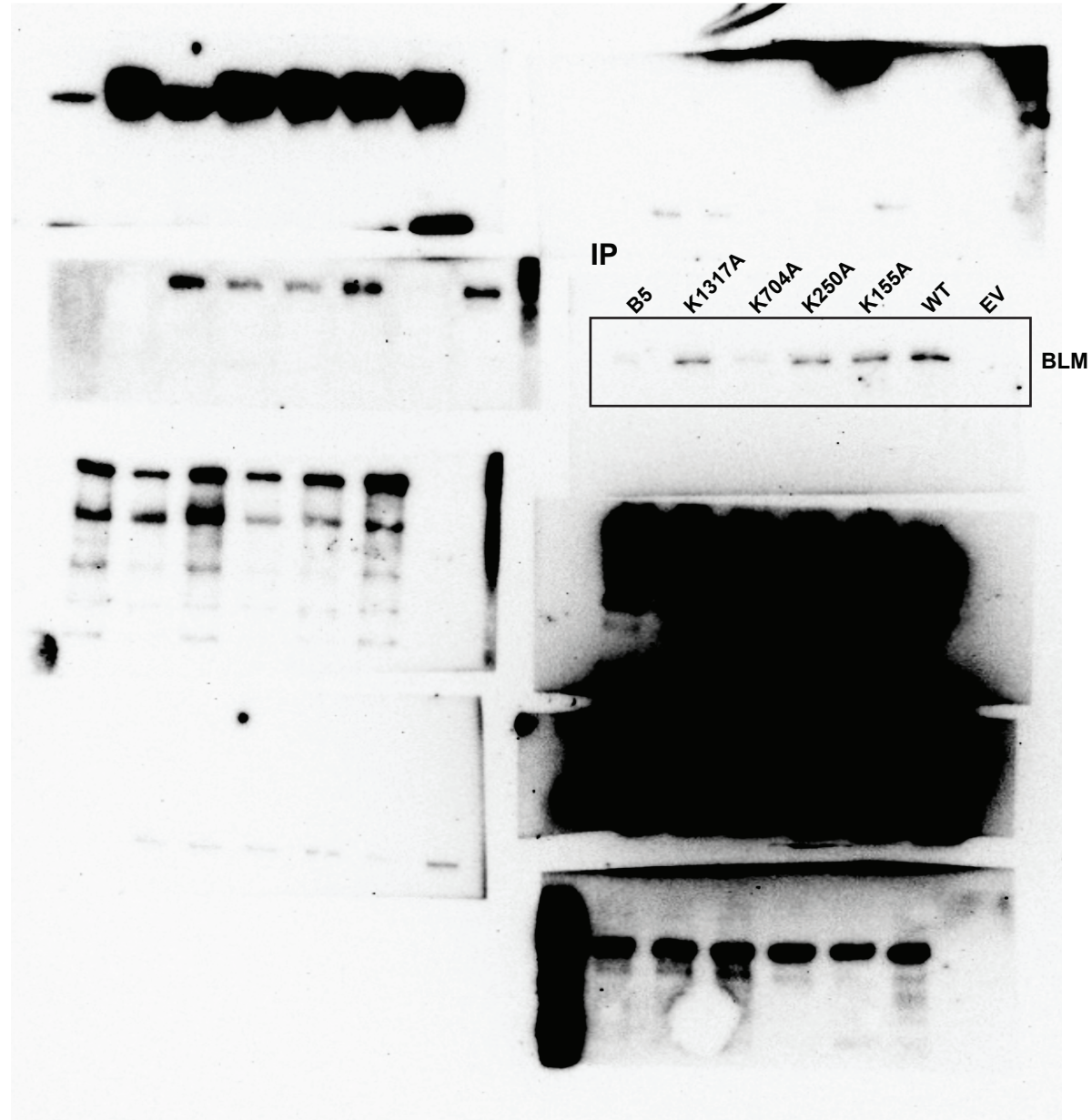

Supplement: Figure 2—figure supplement 3—source data 1. [file elife-90887-fig2-figsupp3-data1.zip › Figure 2-figure supplement_3_souece_data/Figure 2-figure supplement 3_source_data_26.pdf]

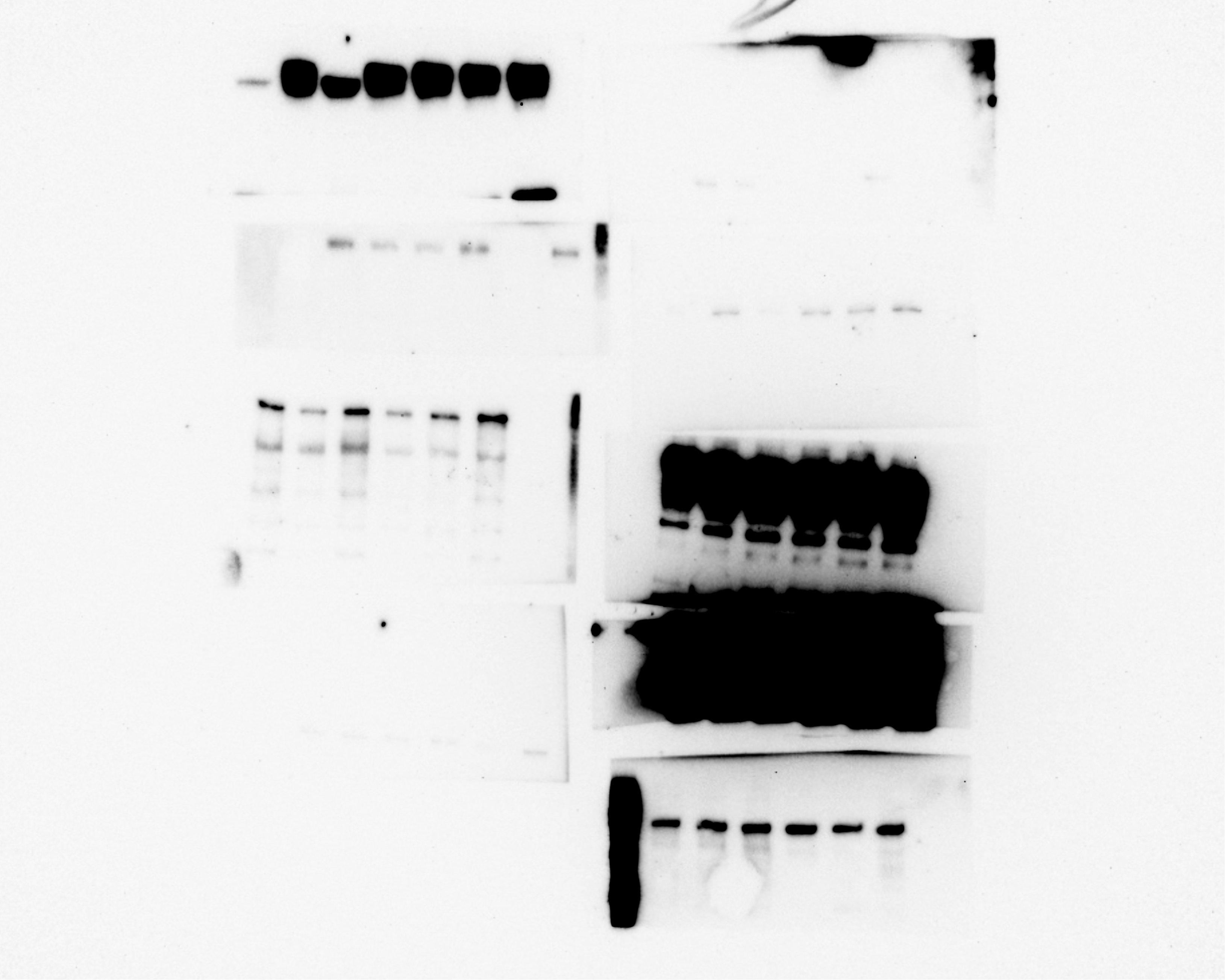

Supplement: Figure 2—figure supplement 3—source data 1. [file elife-90887-fig2-figsupp3-data1.zip › Figure 2-figure supplement_3_souece_data/Figure 2-figure supplement 3_source_data_27.tif]

Figure 2-figure supplement 3\_source\_data\_28

A

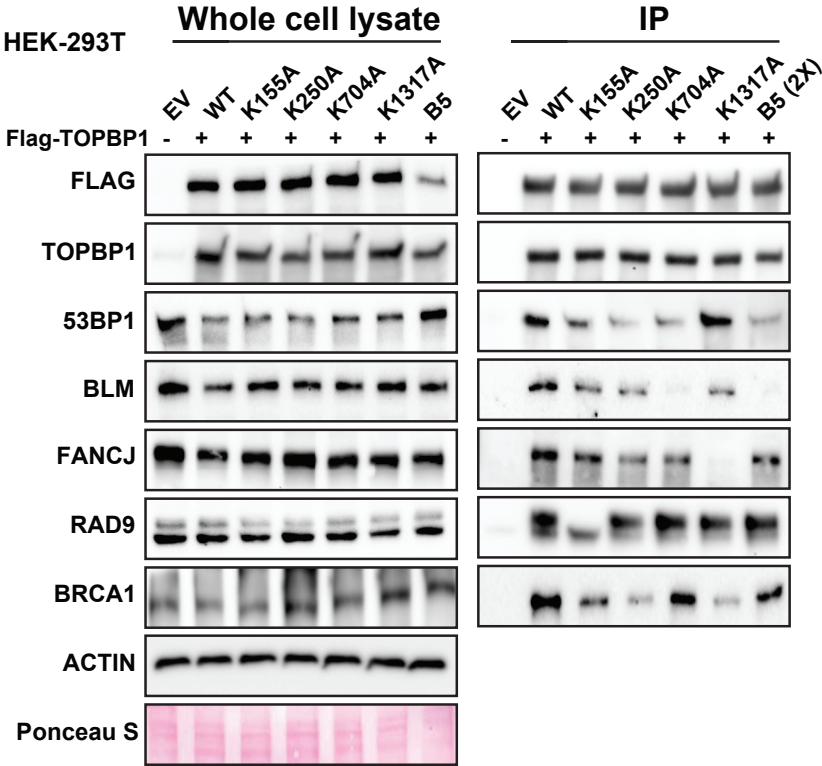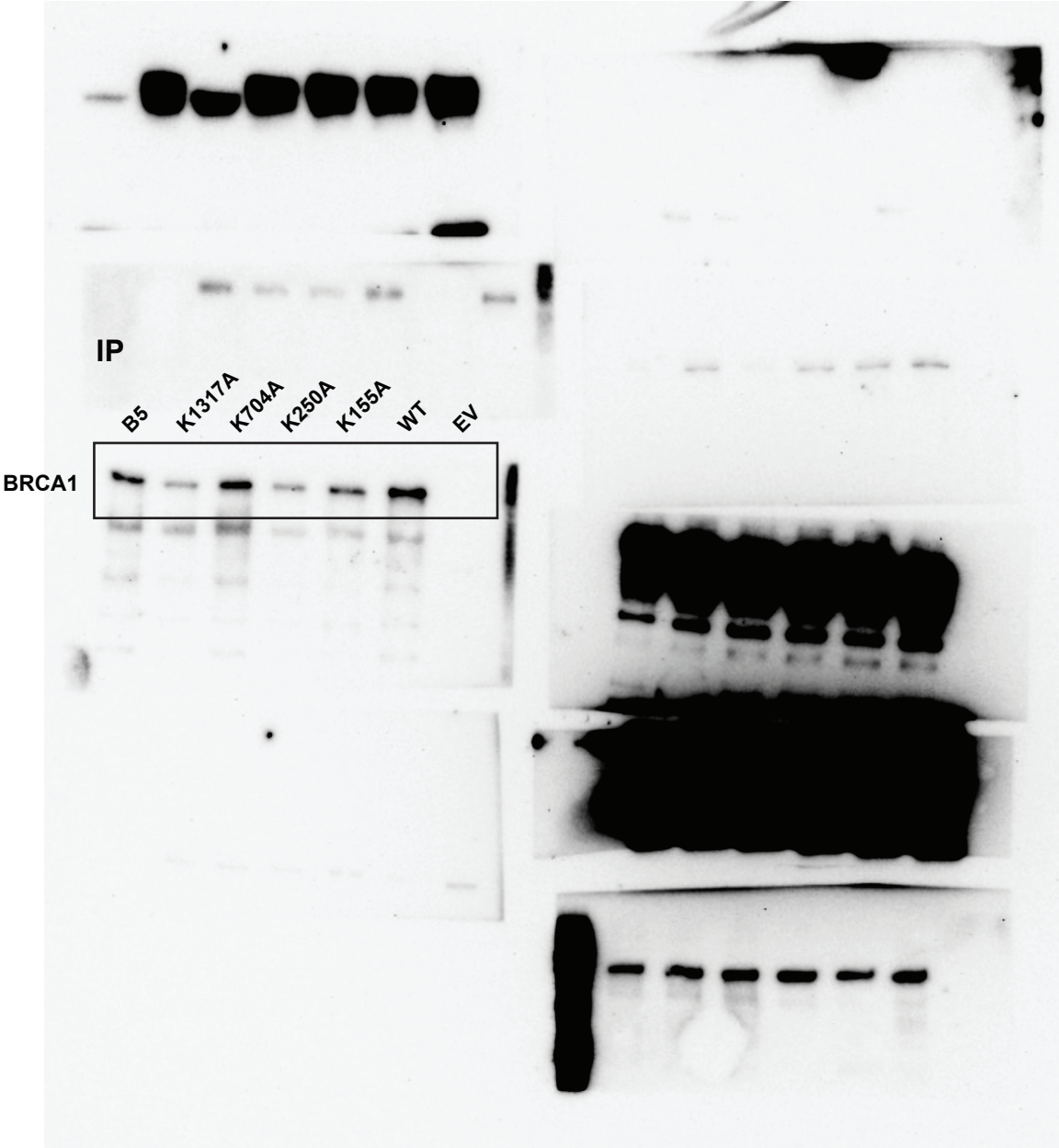

Supplement: Figure 2—figure supplement 3—source data 1. [file elife-90887-fig2-figsupp3-data1.zip › Figure 2-figure supplement_3_souece_data/Figure 2-figure supplement 3_source_data_28.pdf]

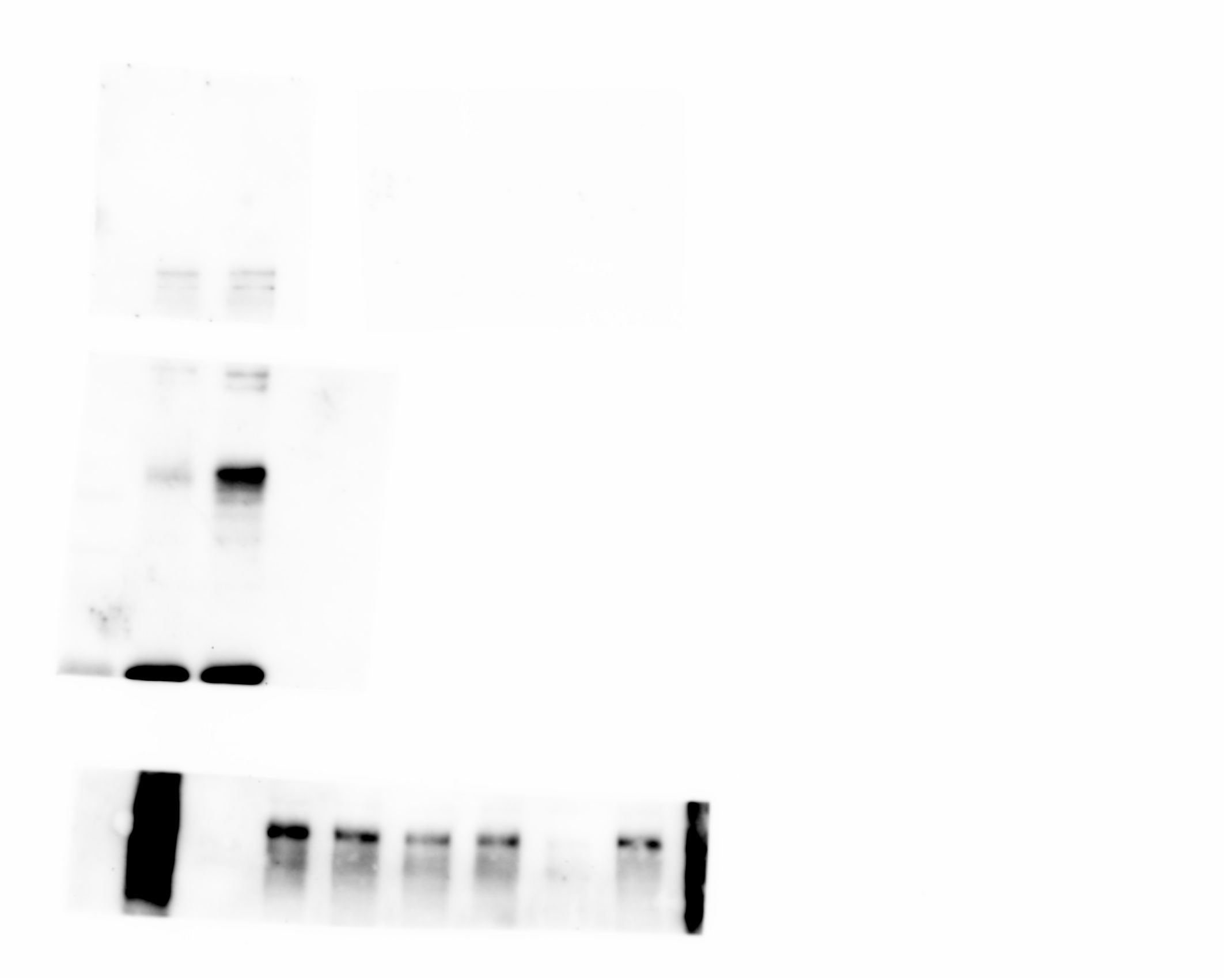

Supplement: Figure 2—figure supplement 3—source data 1. [file elife-90887-fig2-figsupp3-data1.zip › Figure 2-figure supplement_3_souece_data/Figure 2-figure supplement 3_source_data_29.tif]

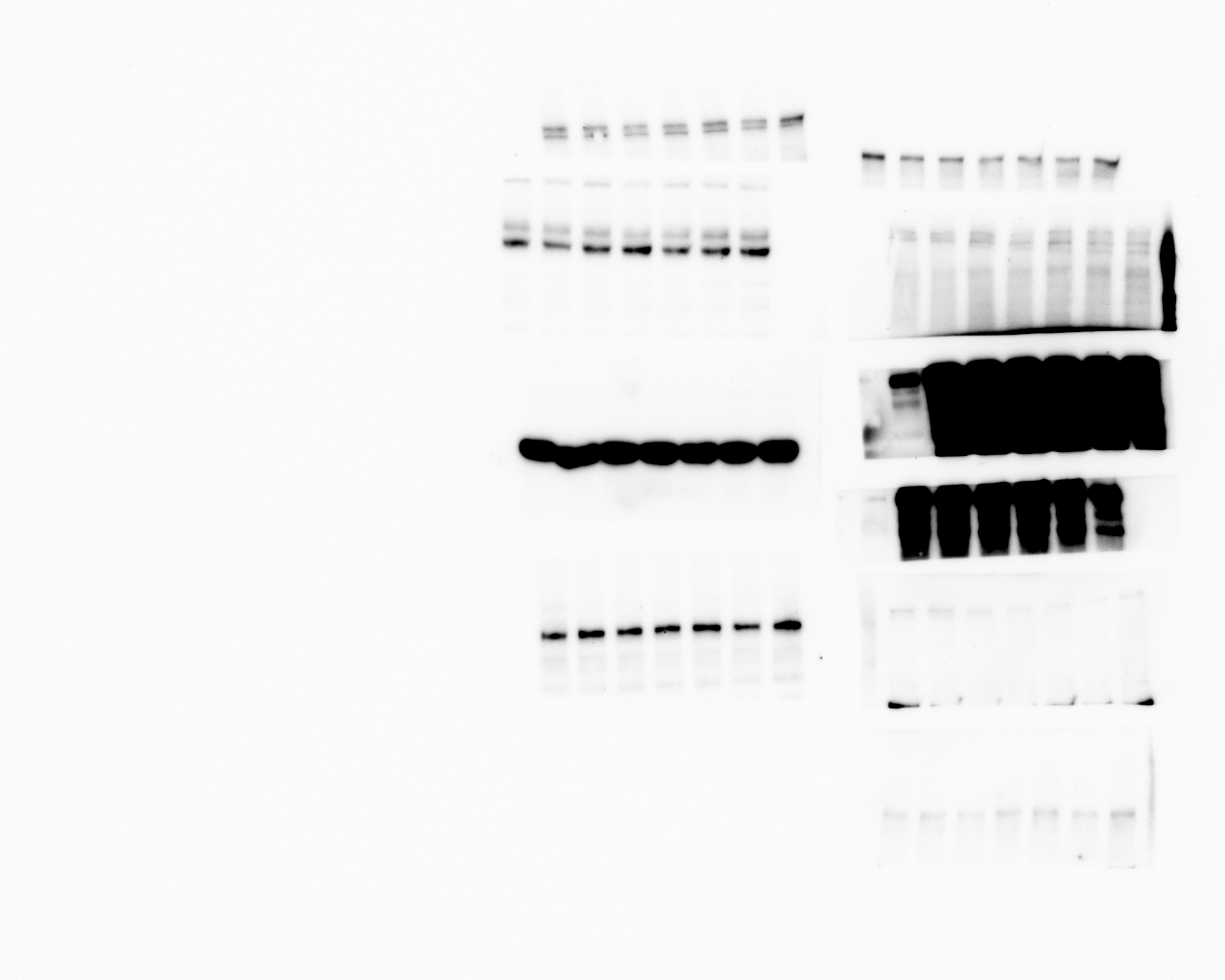

Supplement: Figure 2—figure supplement 3—source data 1. [file elife-90887-fig2-figsupp3-data1.zip › Figure 2-figure supplement_3_souece_data/Figure 2-figure supplement 3_source_data_3.tif]

Figure 2-figure supplement 3\_source\_data\_30

A

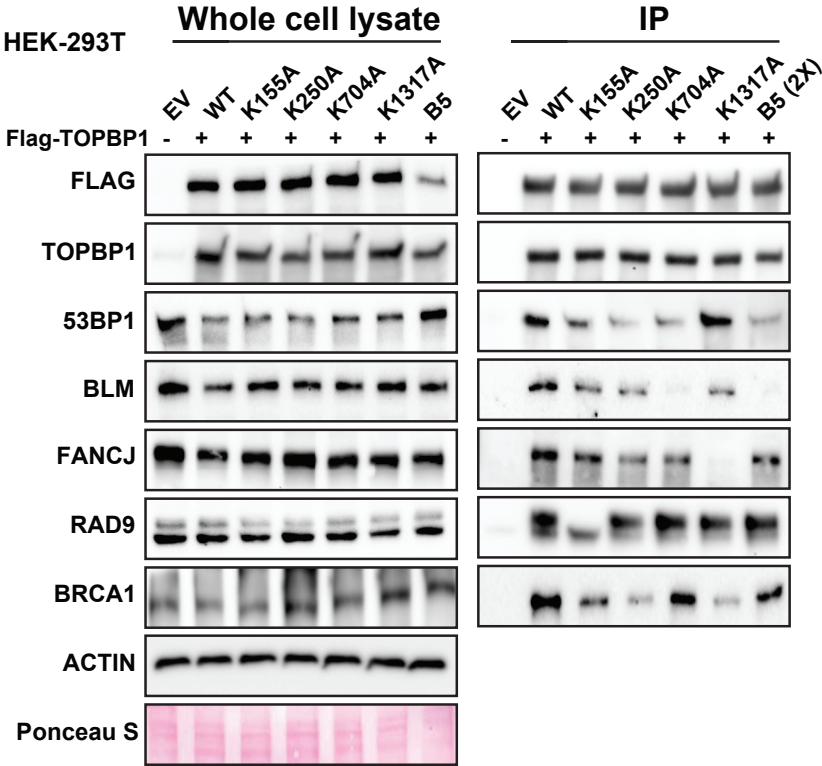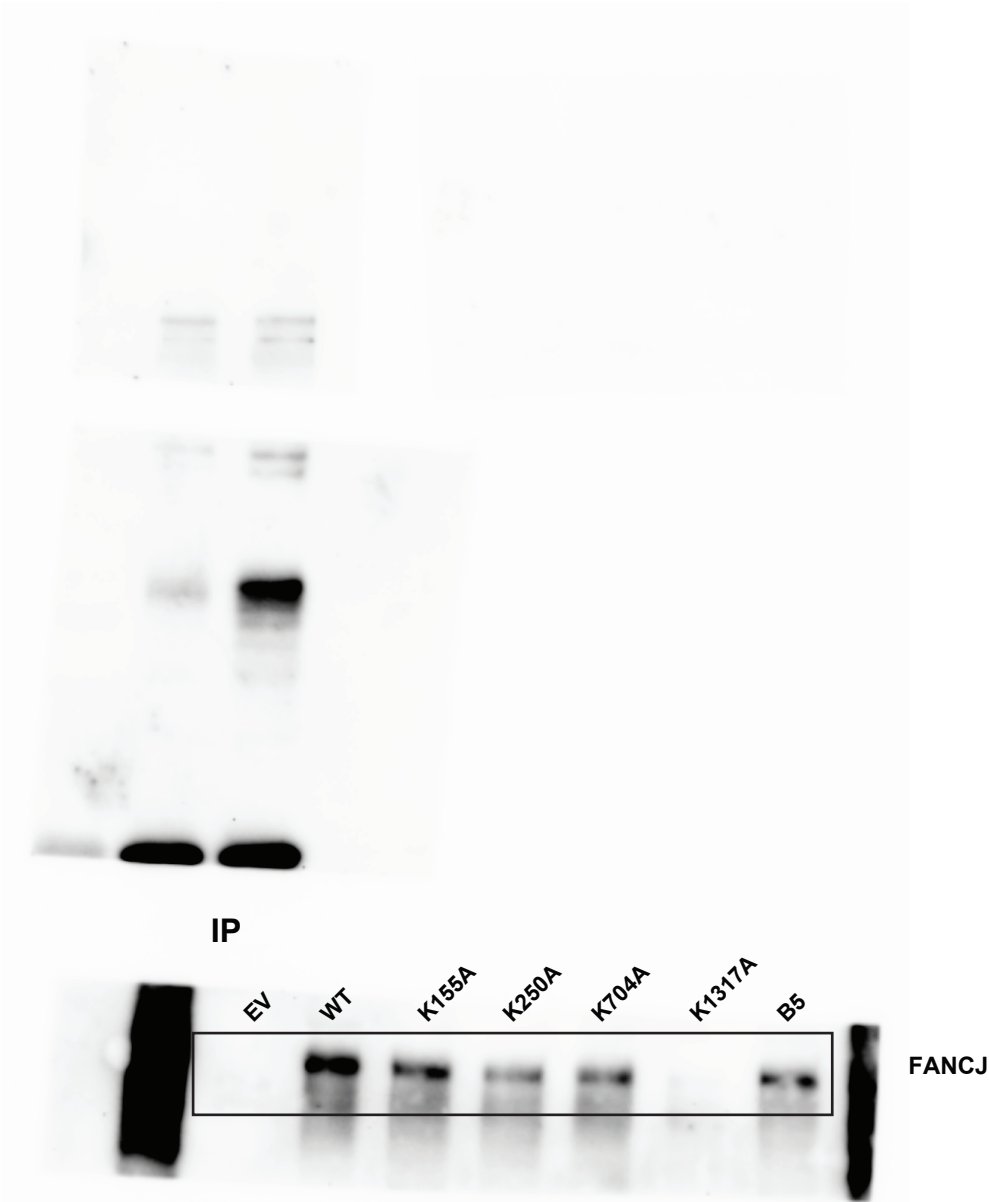

Supplement: Figure 2—figure supplement 3—source data 1. [file elife-90887-fig2-figsupp3-data1.zip › Figure 2-figure supplement_3_souece_data/Figure 2-figure supplement 3_source_data_30.pdf]

Figure 2-figure supplement 3\_source\_data\_4

A

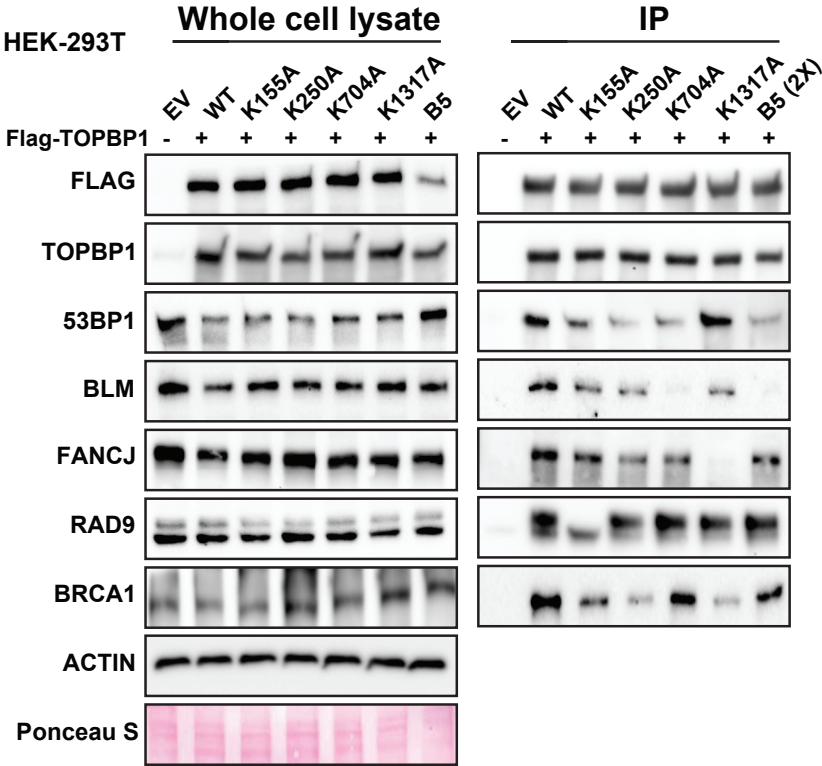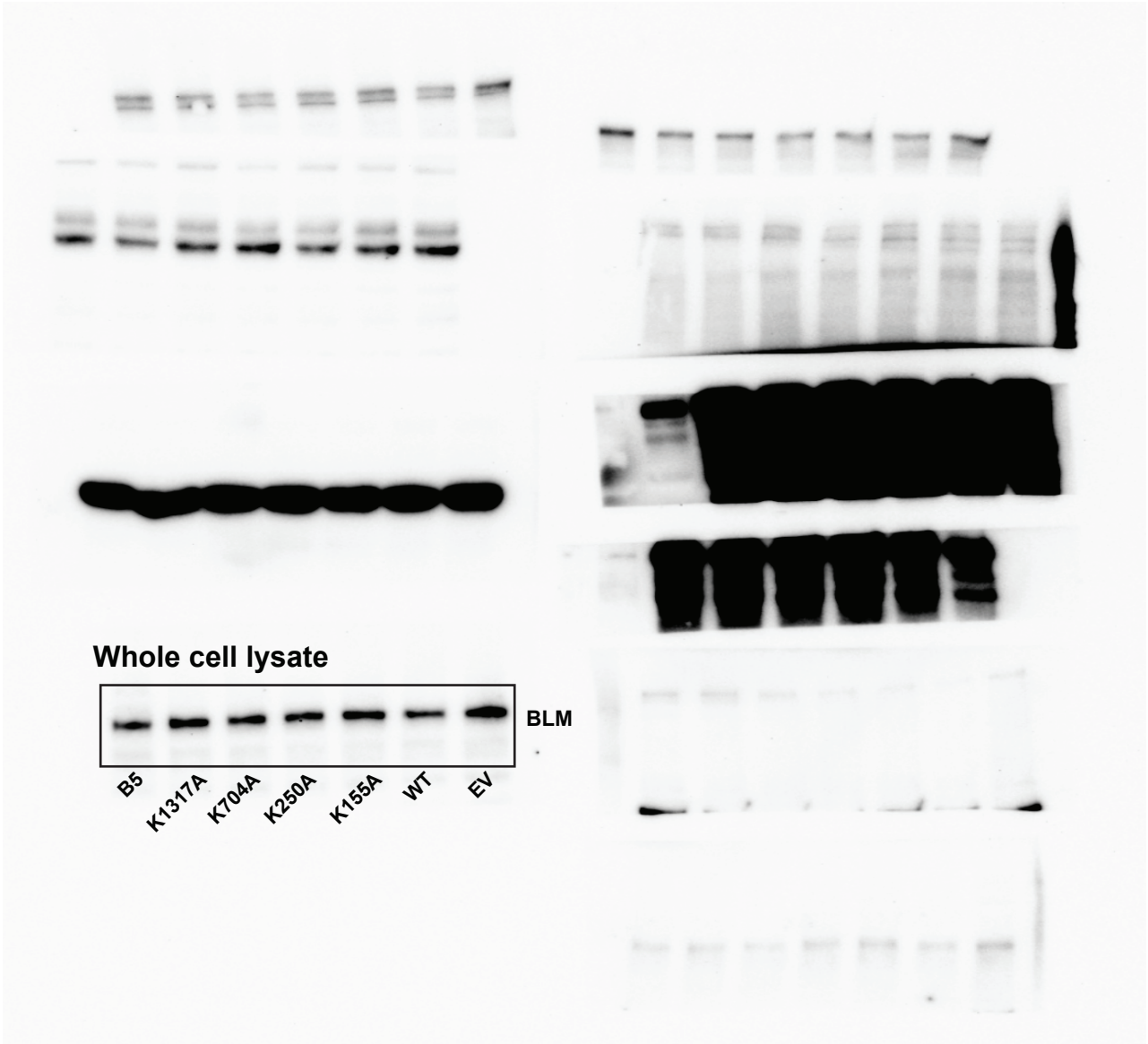

Supplement: Figure 2—figure supplement 3—source data 1. [file elife-90887-fig2-figsupp3-data1.zip › Figure 2-figure supplement_3_souece_data/Figure 2-figure supplement 3_source_data_4.pdf]

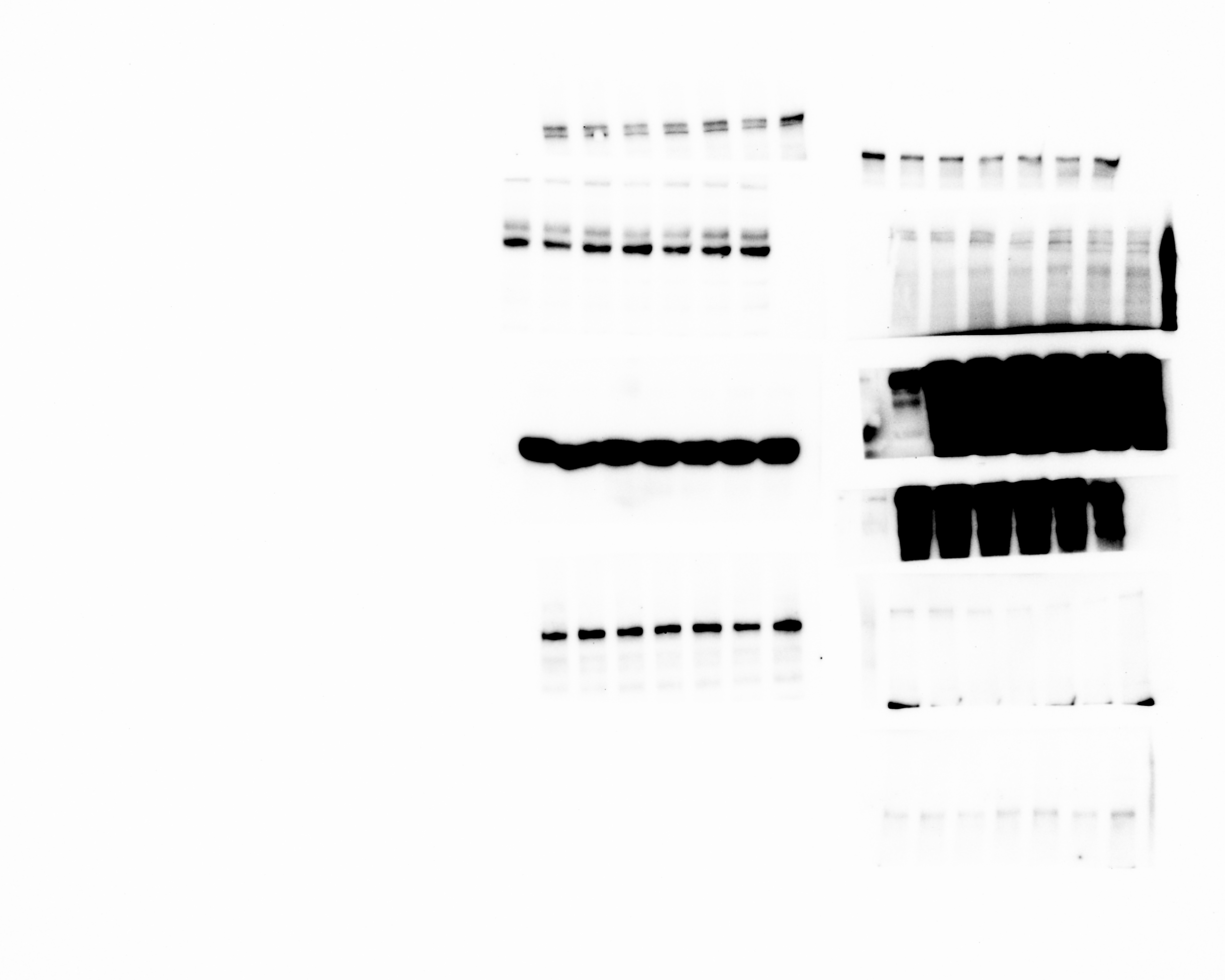

Supplement: Figure 2—figure supplement 3—source data 1. [file elife-90887-fig2-figsupp3-data1.zip › Figure 2-figure supplement_3_souece_data/Figure 2-figure supplement 3_source_data_5.tif]

Figure 2-figure supplement 3\_source\_data\_6

A

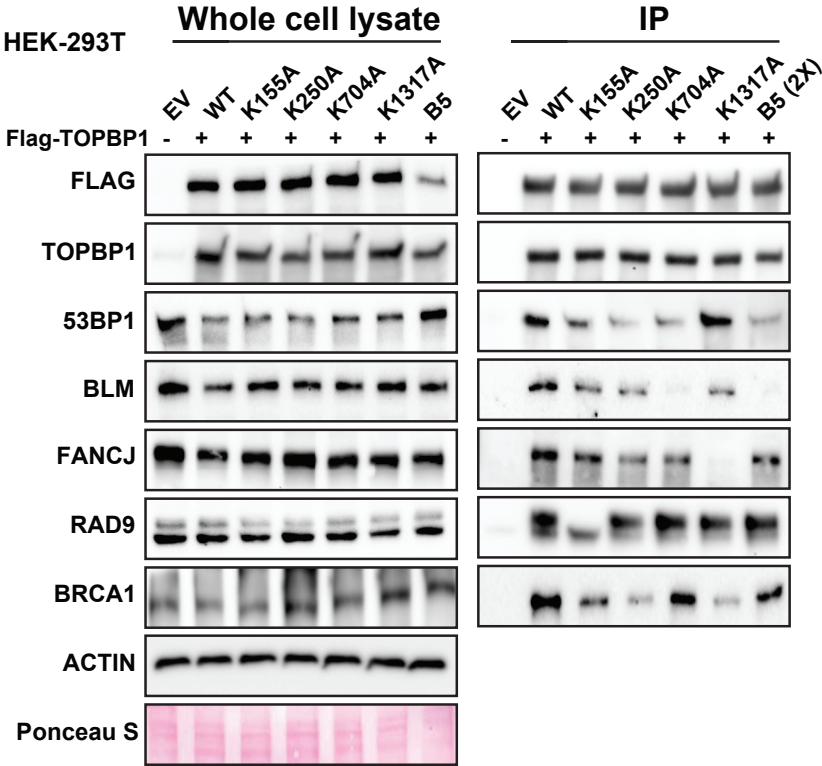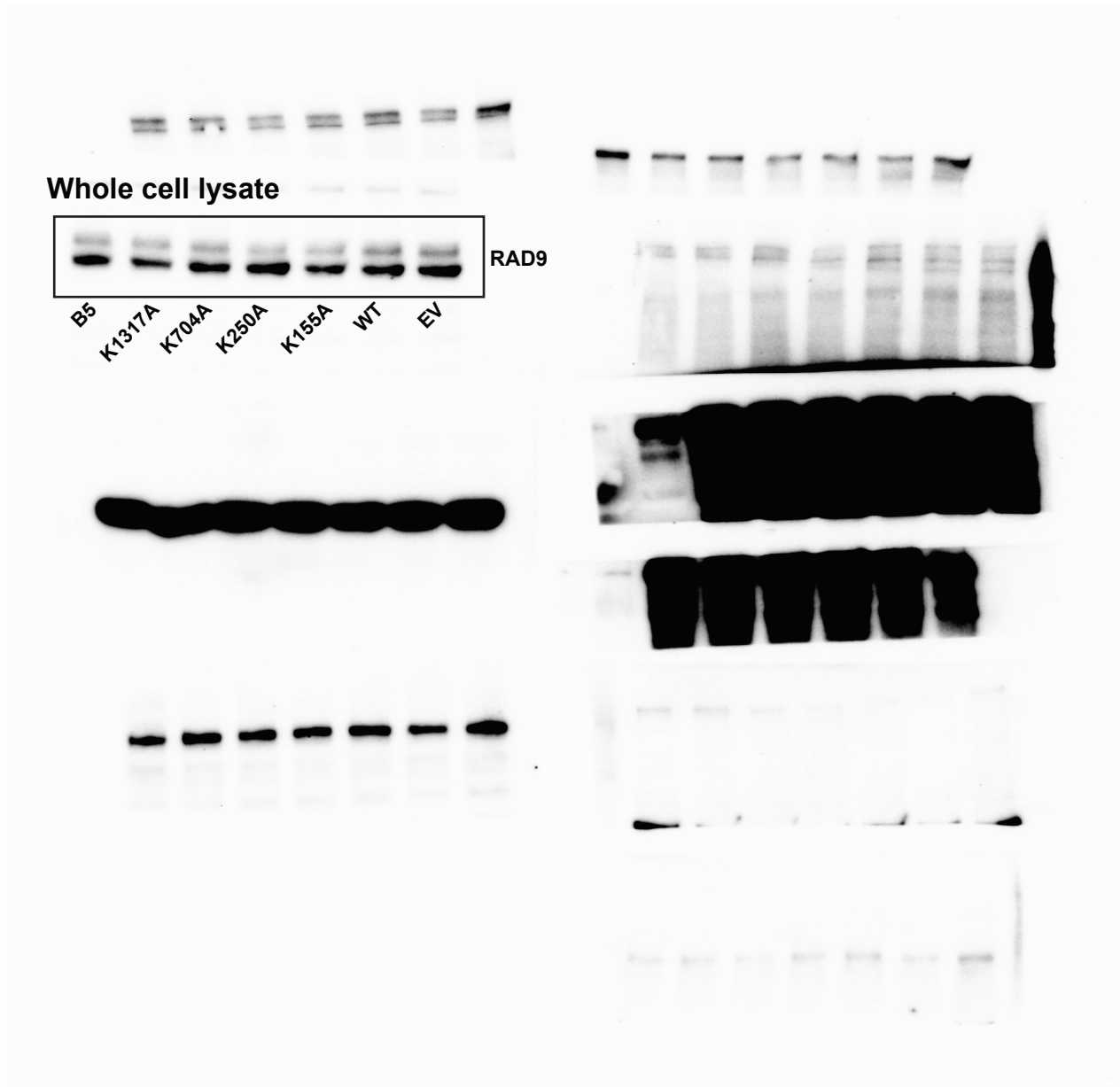

Supplement: Figure 2—figure supplement 3—source data 1. [file elife-90887-fig2-figsupp3-data1.zip › Figure 2-figure supplement_3_souece_data/Figure 2-figure supplement 3_source_data_6.pdf]

Figure 2-figure supplement 3\_source\_data\_8

A

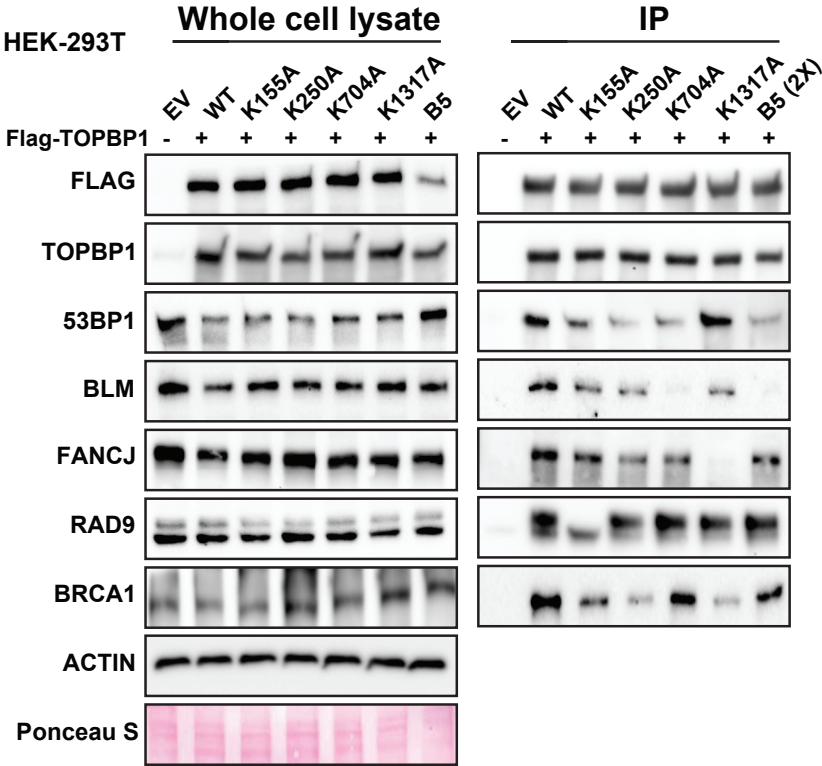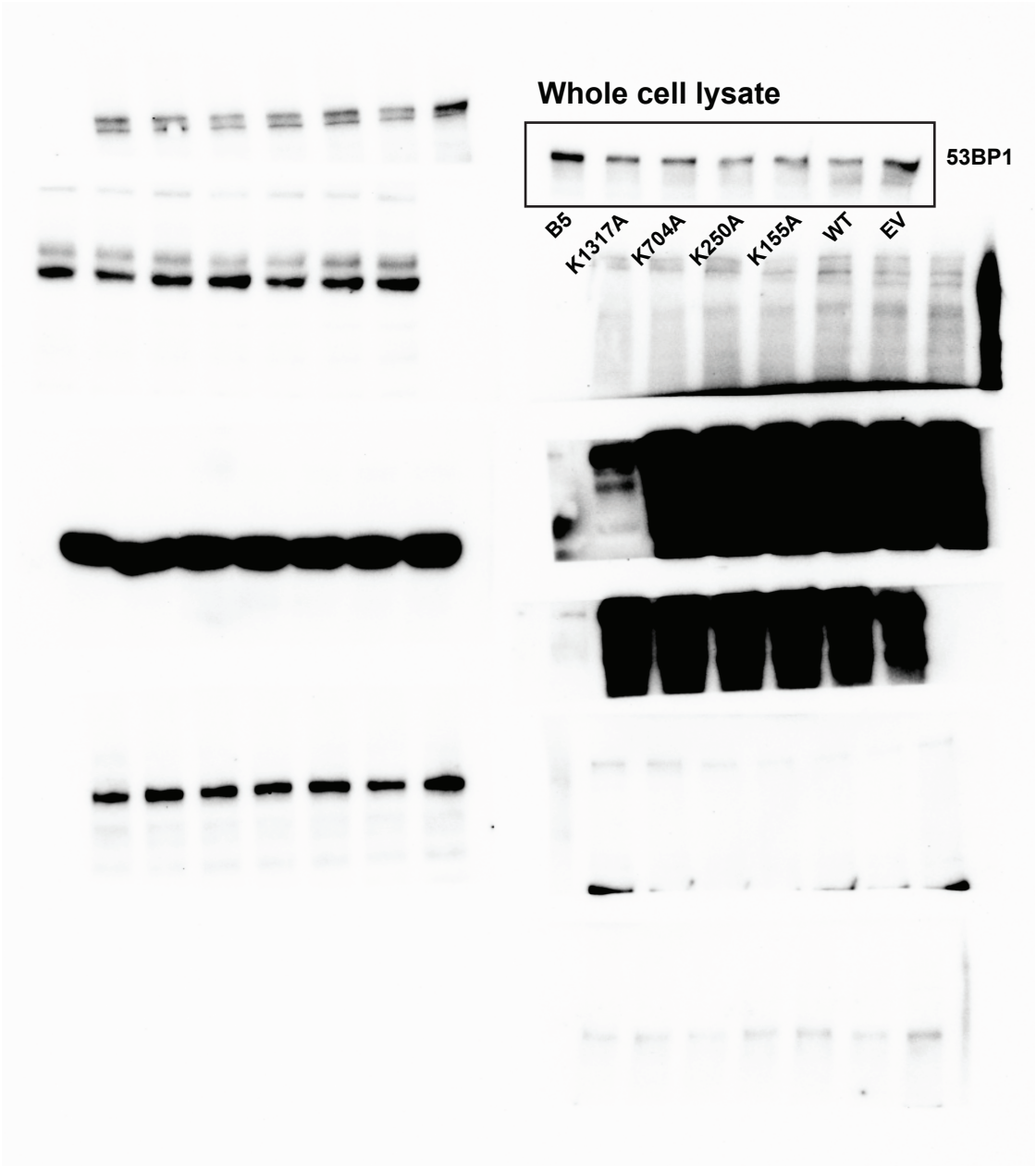

Supplement: Figure 2—figure supplement 3—source data 1. [file elife-90887-fig2-figsupp3-data1.zip › Figure 2-figure supplement_3_souece_data/Figure 2-figure supplement 3_source_data_8.pdf]

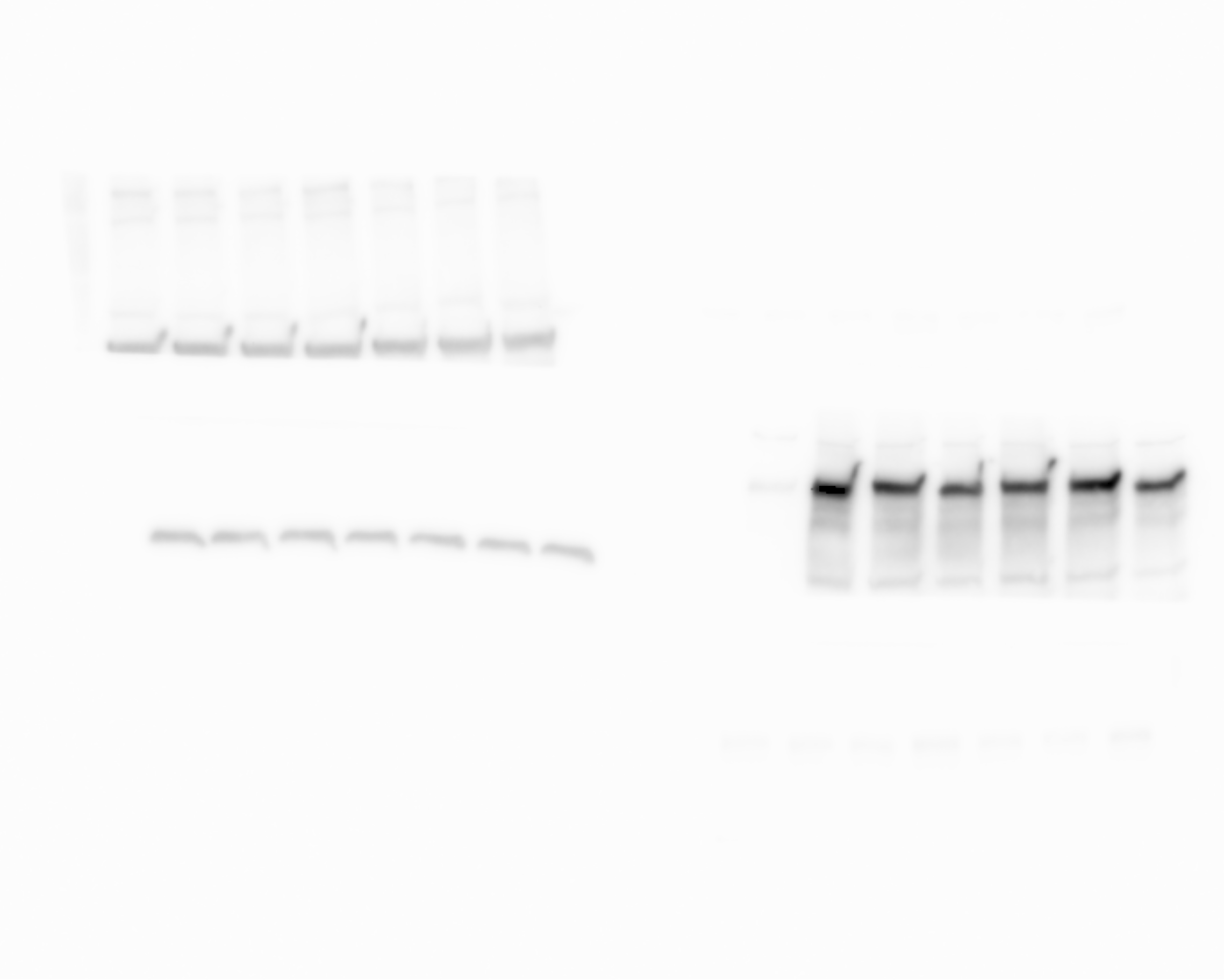

Supplement: Figure 2—figure supplement 3—source data 1. [file elife-90887-fig2-figsupp3-data1.zip › Figure 2-figure supplement_3_souece_data/Figure 2-figure supplement 3_source_data_9.tif]

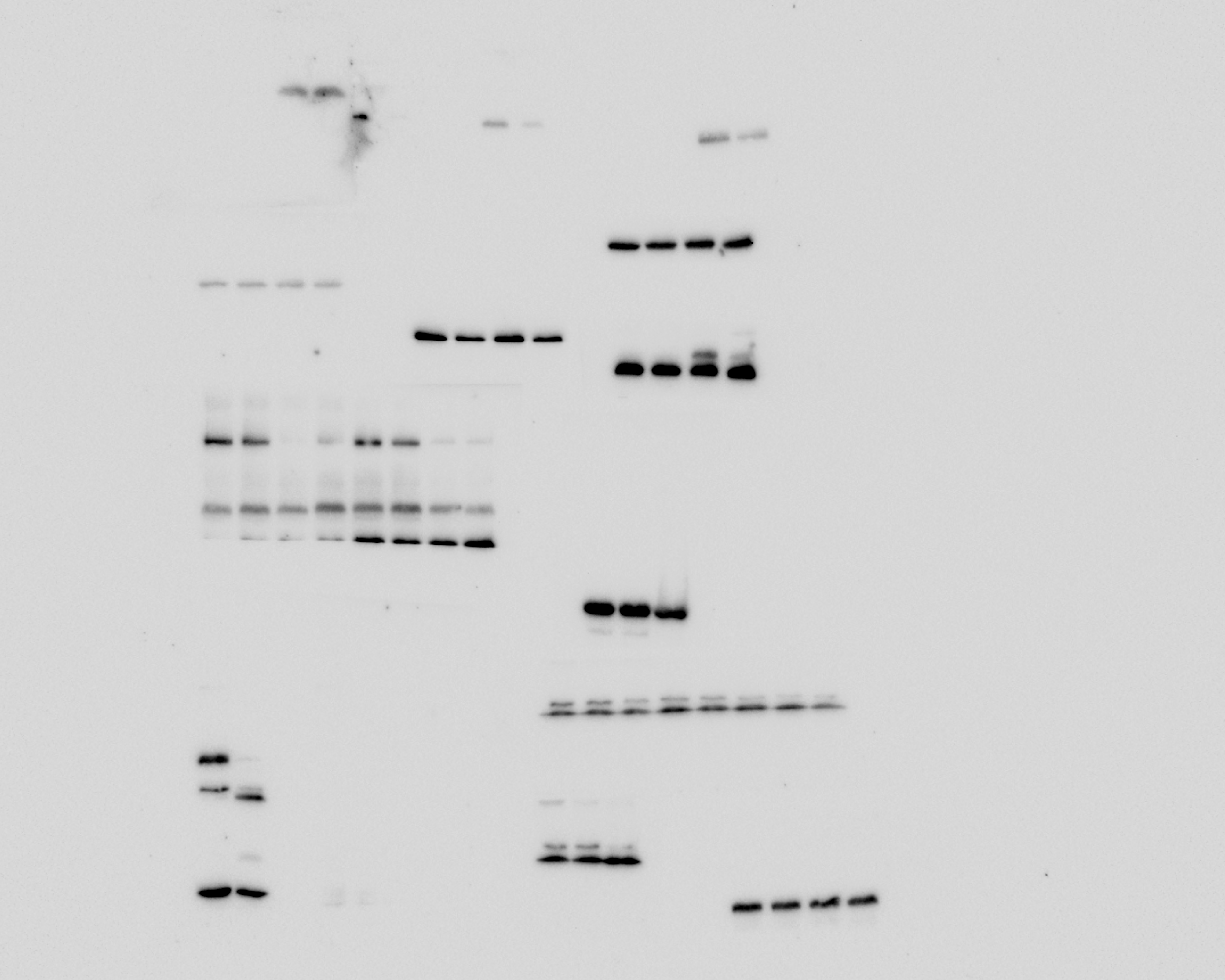

Supplement: Figure 2—figure supplement 4—source data 1. [file elife-90887-fig2-figsupp4-data1.zip › Figure 2-figure supplement 4_source_data_1.tif]

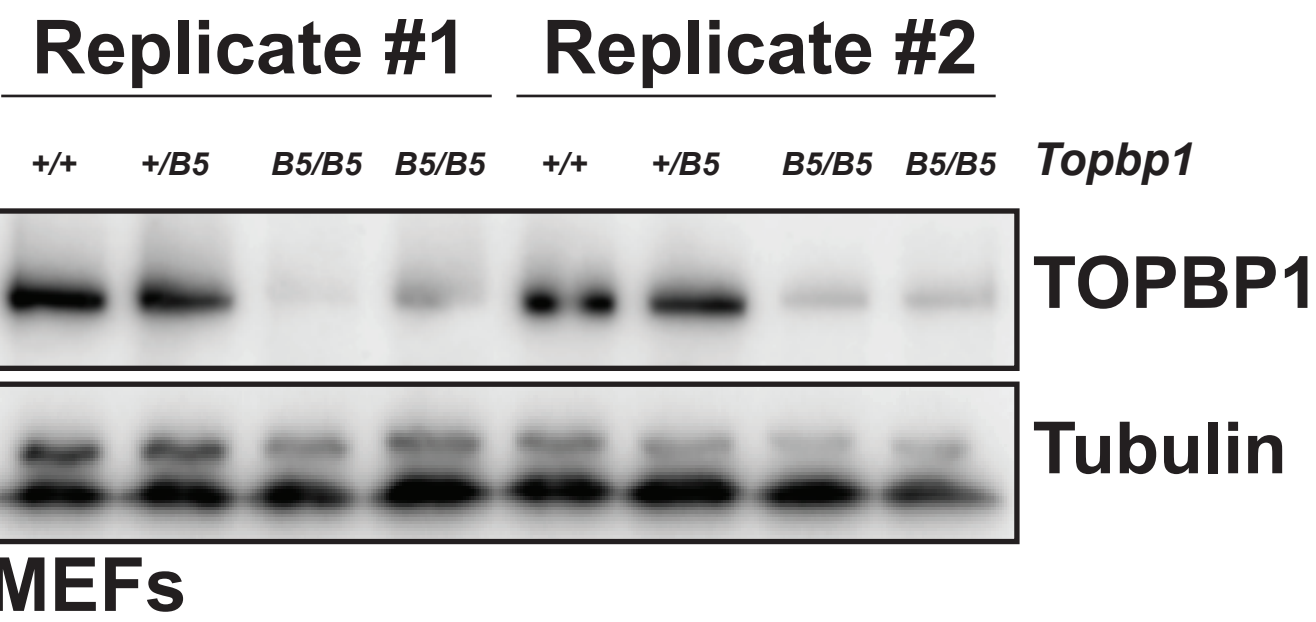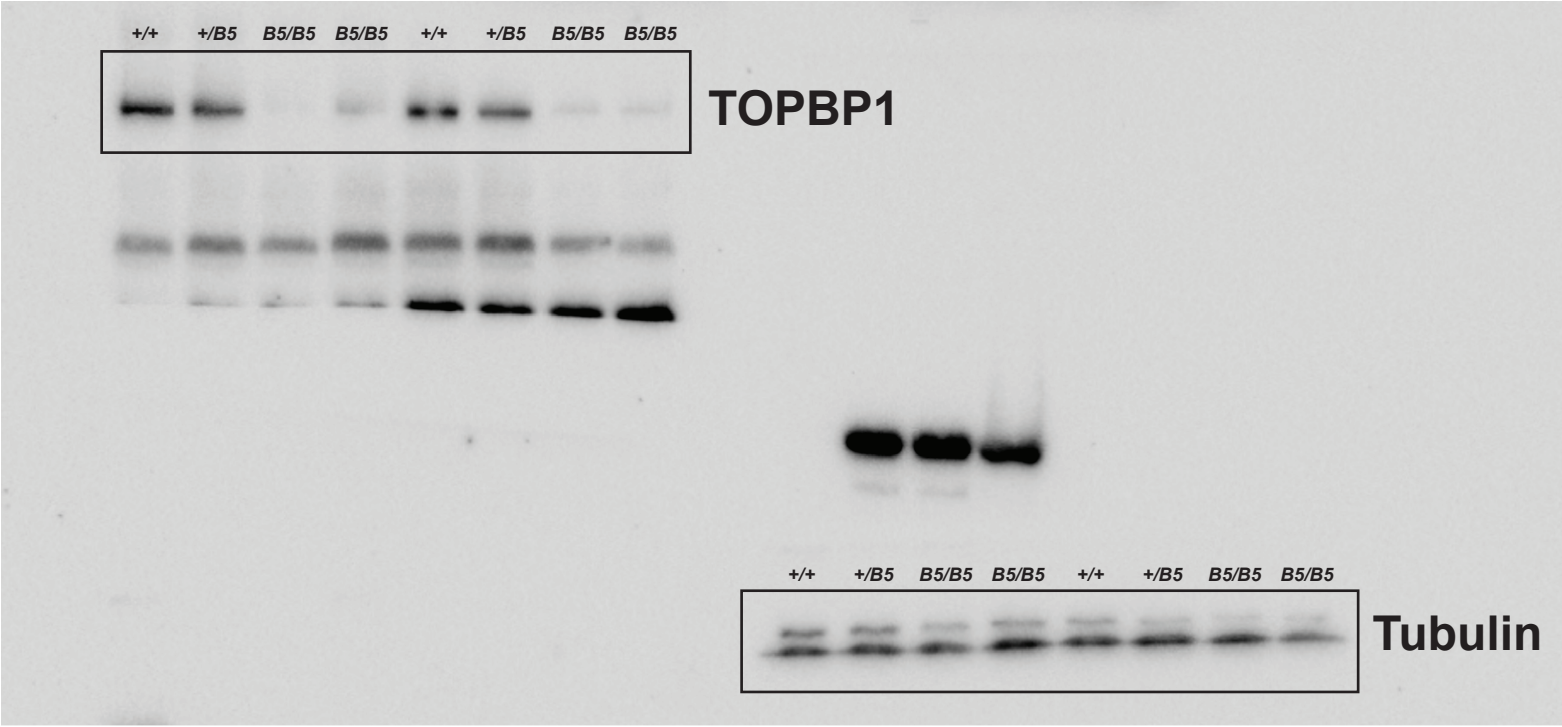

Supplement: Figure 2—figure supplement 4—source data 2. [file elife-90887-fig2-figsupp4-data2.pdf]
