# Supplementary material for: A TOPBP1 allele causing male infertility uncouples XY silencing dynamics from sex body formation
Source: eLife. 2024 Feb 23;12:RP90887. doi: 10.7554/eLife.90887 (PMC10942628; doi:10.7554/eLife.90887)

Figure 2-figure supplement 3_source_data_14

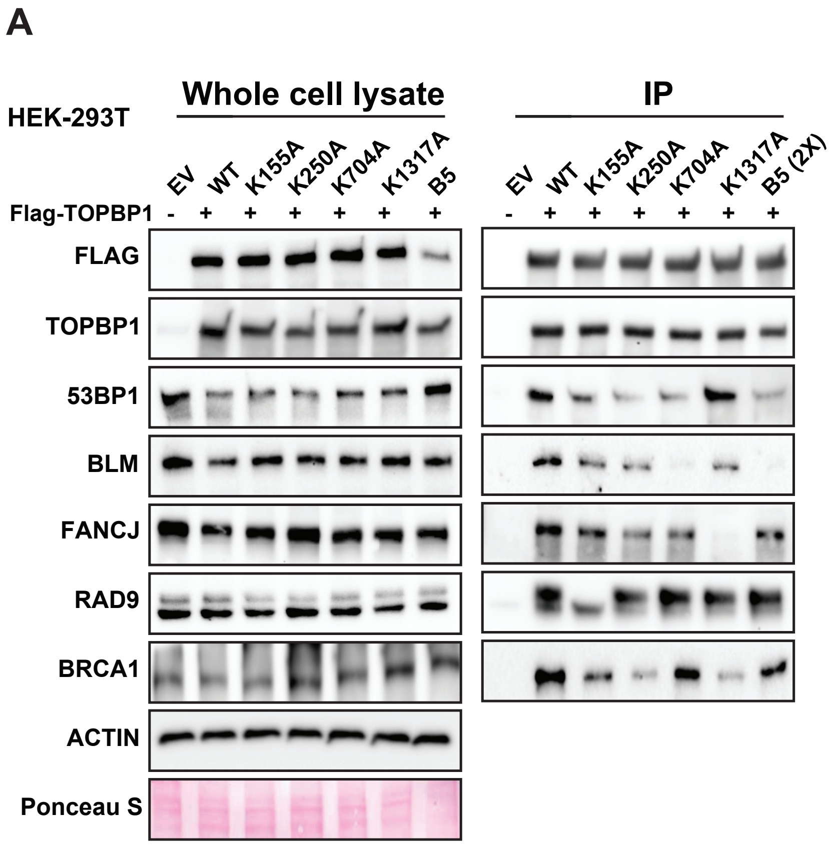

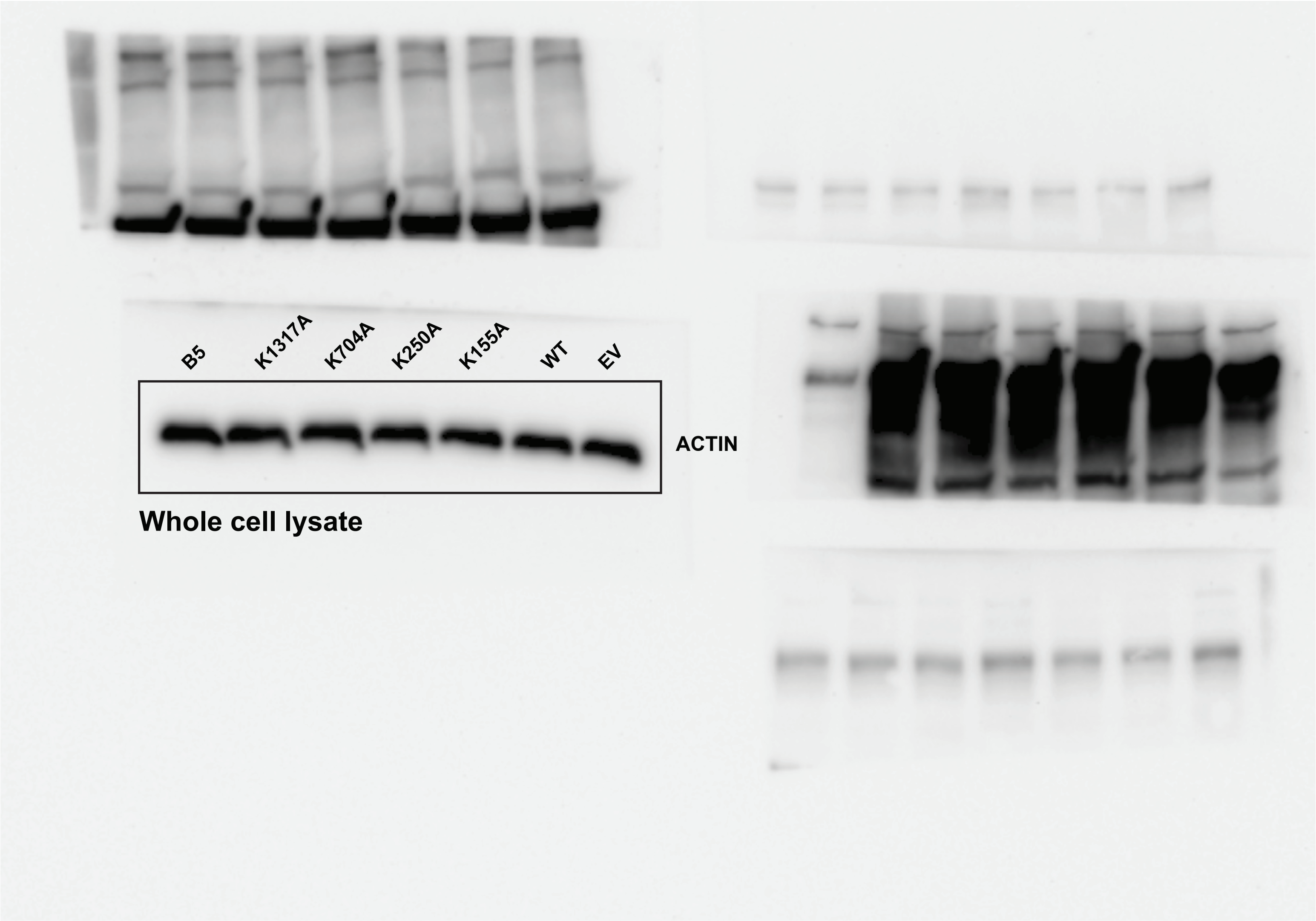

Supplement: Figure 2—figure supplement 3—source data 1. [file elife-90887-fig2-figsupp3-data1.zip › Figure 2-figure supplement_3_souece_data/Figure 2-figure supplement 3_source_data_14.pdf]
