# Supplementary material for: A TOPBP1 allele causing male infertility uncouples XY silencing dynamics from sex body formation
Source: eLife. 2024 Feb 23;12:RP90887. doi: 10.7554/eLife.90887 (PMC10942628; doi:10.7554/eLife.90887)
Supplement: Figure 2—figure supplement 3—source data 1. [file elife-90887-fig2-figsupp3-data1.zip › Figure 2-figure supplement 3 source data titles.docx]

Figure 2-figure supplement 3 source data titles

Figure 2-figure supplement 3-source data 1. Original file for the Western blot analysis for the whole-cell lysate in Figure 2-figure supplement 3A (anti-Flag).

Figure 2-figure supplement 3-source data 2. PDF containing Figure 2-figure supplement 3A and original scans of the relevant Western blot analysis for the whole cell lysate (anti-Flag) with highlighted bands and labels.

Figure 2-figure supplement 3-source data 3. Original file for the Western blot analysis for the whole cell lysate in Figure 2-figure supplement 3A (anti-BLM).

Figure 2-figure supplement 3-source data 4. PDF containing Figure 2-figure supplement 3A and original scans of the relevant Western blot analysis for the whole cell lysate (anti-BLM) with highlighted bands and labels.

Figure 2-figure supplement 3-source data 5. Original file for the Western blot analysis for the whole cell lysate in Figure 2-figure supplement 3A (anti-RAD9

Figure 2-figure supplement 3-source data 6. PDF containing Figure 2-figure supplement 3A and original scans of the relevant Western blot analysis for the whole cell lysate (anti-RAD9) with highlighted bands and labels.

Figure 2-figure supplement 3-source data 7. Original file for the Western blot analysis for the whole cell lysate in Figure 2-figure supplement 3A (anti-53BP1).

Figure 2-figure supplement 3-source data 8. PDF containing Figure 2-figure supplement 3A and original scans of the relevant Western blot analysis for the whole cell lysate (anti-53BP1) with highlighted bands and labels.

Figure 2-figure supplement 3-source data 9. Original file for the Western blot analysis for the whole cell lysate in Figure 2-figure supplement 3A (anti-TOPBP1).

Figure 2-figure supplement 3-source data 10. PDF containing Figure 2-figure supplement 3A and original scans of the relevant Western blot analysis for the whole cell lysate (anti-TOPBP1) with highlighted bands and labels.

Figure 2-figure supplement 3-source data 11. Original file for the Western blot analysis for the whole cell lysate in Figure 2-figure supplement 3A (anti-FANCJ).

Figure 2-figure supplement 3-source data 12. PDF containing Figure 2-figure supplement 3A and original scans of the relevant Western blot analysis for the whole cell lysate (anti-FANCJ) with highlighted bands and labels.

Figure 2-figure supplement 3-source data 13. Original file for the Western blot analysis for the whole cell lysate in Figure 2-figure supplement 3A (anti-ACTIN).

Figure 2-figure supplement 3-source data 14. PDF containing Figure 2-figure supplement 3A and original scans of the relevant Western blot analysis for the whole cell lysate (anti-ACTIN) with highlighted bands and labels.

Figure 2-figure supplement 3-source data 15. Original file for the Western blot analysis for the 1462 whole cell lysate in Figure 2-figure supplement 3A (anti-BRCA1).

Figure 2-figure supplement 3-source data 16. PDF containing Figure 2-figure supplement 3A and original scans of the relevant Western blot analysis for the whole cell lysate (anti-BRCA1) with highlighted bands and labels.

Figure 2-figure supplement 3-source data 17. Original file for the Western blot analysis for the IP in Figure 2-figure supplement 3A (anti-53BP1).

Figure 2-figure supplement 3-source data 18. PDF containing Figure 2-figure supplement 3A and original scans of the relevant Western blot analysis for the IP (anti-53BP1) with highlighted bands and labels.

Figure 2-figure supplement 3-source data 19. Original file for the Western blot analysis for the IP 1 in Figure 2-figure supplement 3A (anti-FLAG).

Figure 2-figure supplement 3-source data 20. PDF containing Figure 2-figure supplement 3A and original scans of the relevant Western blot analysis for the IP (anti-FLAG) with highlighted bands and labels.

Figure 2-figure supplement 3-source data 21. Original file for the Western blot analysis for the IP 1 in Figure 2-figure supplement 3A (anti-TOPBP1).

Figure 2-figure supplement 3-source data 22. PDF containing Figure 2-figure supplement 3A and original scans of the relevant Western blot analysis for the IP (anti-TOPBP1) with highlighted bands and labels.

Figure 2-figure supplement 3-source data 23. Original file for the Western blot analysis for the IP 1 in Figure 2-figure supplement 3A (anti-RAD9).

Figure 2-figure supplement 3-source data 24. PDF containing Figure 2-figure supplement 3A and original scans of the relevant Western blot analysis for the IP (anti-RAD9) with highlighted bands and labels.

Figure 2-figure supplement 3-source data 25. Original file for the Western blot analysis for the IP in Figure 2-figure supplement 3A (anti-BLM).

Figure 2-figure supplement 3-source data 26. PDF containing Figure 2-figure supplement 3A and original scans of the relevant Western blot analysis for the IP (anti-BLM) with highlighted bands and labels.

Figure 2-figure supplement 3-source data 27. Original file for the Western blot analysis for the IP 1 in Figure 2-figure supplement 3A (anti-BRCA1).

Figure 2-figure supplement 3-source data 28. PDF containing Figure 2-figure supplement 3A and original scans of the relevant Western blot analysis for the IP (anti-BRCA1) with highlighted bands and labels.

Figure 2-figure supplement 3-source data 29. Original file for the Western blot analysis for the IP in Figure 2-figure supplement 3A (anti-FANCJ).

Figure 2-figure supplement 3-source data 30. PDF containing Figure 2-figure supplement 3A and original scans of the relevant Western blot analysis for the IP (anti-FANCJ) with highlighted bands and labels.
